# The pro-inflammatory cytokines IFN-α and TNF-α inhibit organoid-derived extravillous trophoblast invasion

**DOI:** 10.1016/j.isci.2026.116431

**Published:** 2026-06-20

**Authors:** A. Jantine van Voorden, Fangxu Lin, Souad Boussata, Remco Keijser, Liana Barenbrug, Bente Horselenberg, Ans M.M. van Pelt, Wendy Dankers, Susana M. Chuva de Sousa Lopes, Gijs B. Afink

**Affiliations:** 1 Reproductive Biology Laboratory, Amsterdam University Medical Center, University of Amsterdam, Meibergdreef 9, 1105 AZ Amsterdam, the Netherlands; 2 Amsterdam Reproduction and Development Research Institute, Amsterdam, the Netherlands; 3 Department of Rheumatology and Clinical Immunology, Amsterdam University Medical Center, University of Amsterdam, Meibergdreef 9, 1105 AZ Amsterdam, the Netherlands; 4 Department of Experimental Immunology, Amsterdam University Medical Center, University of Amsterdam, Meibergdreef 9, 1105 AZ Amsterdam, the Netherlands; 5 Amsterdam Institute for Immunology and Infectious Diseases, Amsterdam, the Netherlands; 6 Department of Anatomy and Embryology, Leiden University Medical Center, 2333 ZC Leiden, the Netherlands; 7 The Novo Nordisk Foundation Center for Stem Cell Medicine (reNEW), Leiden University Medical Center, 2333 ZC Leiden, the Netherlands

**Keywords:** molecular biology, immunology, developmental biology

## Abstract

Proper placental development requires extravillous trophoblast (EVT) differentiation and invasion into the maternal decidua to remodel spiral arteries and support fetal growth. Disruptions in these processes contribute to preeclampsia and fetal growth restriction. These pregnancy complications are common in women with immune-mediated inflammatory diseases, which are characterized by elevated levels of pro-inflammatory cytokines such as interferon-α (IFN-α) and tumor necrosis factor-α (TNF-α). However, the direct effects of these cytokines on EVTs remain unclear. Using human trophoblast organoid models, we demonstrate that IFN-α and TNF-α impair EVT invasion while preserving differentiation capacity. High-resolution imaging of untreated organoid-decidua co-cultures revealed extensive trophoblast invasion into decidual stroma and arteries, but invasion was substantially reduced particularly upon IFN-α treatment. Transcriptome profiling identified changes in several invasion-related pathways after cytokine exposure. These findings suggest that elevated IFN-α and TNF-α can directly impair trophoblast invasive capacity, potentially contributing to suboptimal placental development in inflammatory disorders.

## Highlights

• IFN-α/TNF-α reduce extravillous trophoblast invasion despite normal differentiation
• The cytokines impair trophoblast invasion in organoid and decidua co-culture models
• IFN-α/TNF-α alter the expression of invasion-related pathways and key pro-invasive genes
• Reduced trophoblast invasion may underlie adverse outcomes in lupus pregnancies

## Graphical abstract

**Figure:**
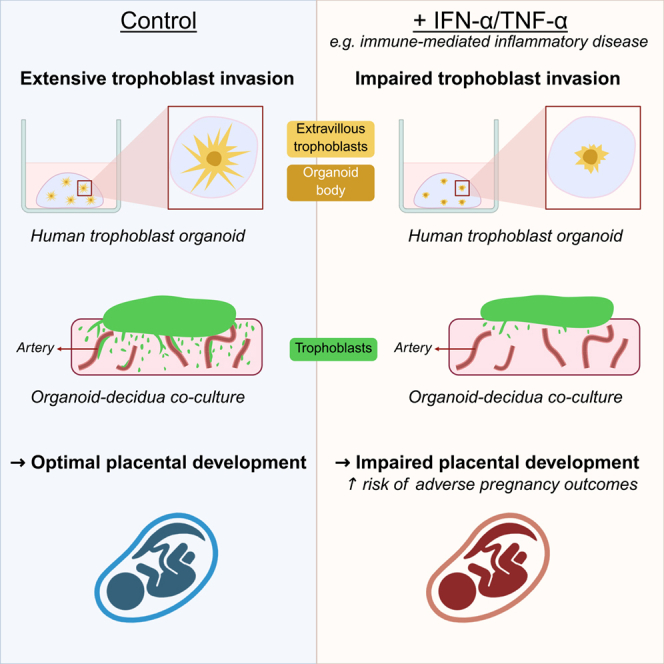

## Introduction

Severe pregnancy complications, including early-onset preeclampsia and fetal growth restriction, are primarily caused by defective placenta development.^1^^,^^2^ The placenta provides the fetus with oxygen and nutrients, and eliminates waste products. Maternal-fetal exchange is mediated by syncytiotrophoblasts (STBs), multinucleated cells derived from the fusion of cytotrophoblasts, which are in direct contact with the maternal blood. At the tips of anchoring placental villi, cytotrophoblasts differentiate into extravillous trophoblasts (EVTs), a process that involves epithelial-mesenchymal transition (EMT), and results in an invasive cell type.^1^^,^^2^^,^^3^^,^^4^^,^^5^ Subsequently, EVTs infiltrate the maternal decidua and actively remodel the spiral arteries, converting them into high-conductance, low-resistance blood vessels. Spiral artery remodeling is characterized by loss of smooth muscle cells and replacement of endothelial cells by EVTs, and is regulated by EVTs as well as decidual natural killer cells.^3^^,^^6^ EVTs can reach the spiral arteries by migration through the decidual stroma (interstitial EVTs) or migration directly from the anchoring villi into the arteries (endovascular EVTs). The processes of EVT differentiation, invasion, and spiral artery remodeling are essential for facilitating an adequate uteroplacental blood flow and proper maternal-fetal exchange; a dysregulation in these processes can result in adverse pregnancy outcomes.^1^^,^^2^^,^^3^^,^^7^^,^^8^^,^^9^

The maternal immune system plays an important role throughout pregnancy, and needs to adapt to the developing embryo and later fetus.^10^^,^^11^ During the peri-implantation period, a pro-inflammatory microenvironment (Th1/Th17 response) is required to allow blastocyst implantation. However, to avoid the rejection of the semi-allogeneic fetus, an anti-inflammatory microenvironment (Th2/regulatory T cell response) becomes dominant during further fetal development. At the end of pregnancy, a pro-inflammatory microenvironment is again established to prepare for parturition. These shifts are tightly regulated by immune cells through the secretion of both pro-inflammatory and anti-inflammatory cytokines. A disturbed immune balance is associated with pregnancy complications, in particular preeclampsia, which is accompanied by systemic inflammation and endothelial cell dysfunction.^10^^,^^11^^,^^12^^,^^13^^,^^14^

Chronic diseases in which the maternal immune system is inherently dysregulated, such as immune-mediated inflammatory diseases, pose an increased risk for adverse pregnancy outcomes.^15^^,^^16^^,^^17^ These diseases are a clinically heterogeneous group of conditions typically characterized by chronic inflammation and organ damage, which are caused or accompanied by overactivity of pro-inflammatory cytokines.^18^^,^^19^ Immune-mediated inflammatory diseases especially affect women, many of whom are of reproductive age, emphasizing the relevance of their effect on pregnancy outcomes. However, it remains unclear whether the involved cytokines directly affect early placenta development, and whether that contributes to the increased risk for pregnancy complications.

Systemic lupus erythematosus (SLE) is an immune-mediated inflammatory disease that is characterized by high activity of type I interferons (IFNs), in particular interferon-α (IFN-α).^20^^,^^21^ Women with SLE have an increased risk for adverse pregnancy outcomes, including preeclampsia, preterm birth, and low birth weight.^16^^,^^22^ In addition, SLE pregnancies are associated with lower placental weight and abnormalities in placental vascularity.^23^ Moreover, increased IFN-α activity correlates with the onset of preeclampsia in patients with SLE,^22^^,^^23^^,^^24^ and elevated IFN-α plasma levels have been associated with lower birth weight.^25^ As IFNs play an important role in the response to viral and bacterial infections, they are crucial for the protection of the fetus from pathogens in normal pregnancies.^26^ However, the effect of increased IFN-α levels on early placentation events has been poorly studied. IFN-β, another type I IFN, was shown to inhibit the fusion of trophoblasts into STBs in a study using the BeWo trophoblast cell line.^27^ In addition, a recent publication showed that IFN-β inhibits EVT invasion by targeting EMT, in an organ-on-a-chip model using primary trophoblasts and endothelial cells.^28^ As IFN-α and IFN-β bind to the same receptor, it is plausible that IFN-α has a similar effect on trophoblasts.

Tumor necrosis factor alpha (TNF-α) is a pro-inflammatory cytokine that is highly secreted in several immune-mediated inflammatory diseases, including SLE, rheumatoid arthritis, psoriasis, and inflammatory bowel disease.^29^^,^^30^ Aberrant levels of TNF-α during pregnancy are associated with preeclampsia and other adverse pregnancy outcomes.^31^ In pregnant mice, TNF-α infusion induces hypertension and proteinuria, resembling preeclampsia.^32^ Several studies using first-trimester placental explants, primary EVTs or trophoblast cell lines, have suggested that TNF-α inhibits EVT invasion, by targeting processes such as extracellular matrix degradation or adhesion, apoptosis, proliferation and/or differentiation.^33^^,^^34^^,^^35^^,^^36^^,^^37^^,^^38^^,^^39^^,^^40^ However, the exact mechanisms remain unclear.

Our possibilities to study early placenta development *in vitro* have increased since the derivation of human trophoblast stem cells (TSCs) from first-trimester placental tissue^41^ and the generation of trophoblast organoids (TOs).^42^^,^^43^ In contrast to other model systems, such as choriocarcinoma cell lines and primary cells, TSCs have the capacity of long-term self-renewal and differentiation into hormone-producing STBs and invasive EVTs.^41^ Moreover, they fulfill the additional criteria for bona fide TSCs as proposed by Lee et al.^44^ and Karvas et al*.*^45^ By seeding TSCs into an extracellular matrix, they self-organize into three-dimensional structures, consisting of a cytotrophoblast layer partly filled with STBs, and can be induced to give rise to EVTs.^46^ Because of their invasive nature, the EVTs invade the extracellular matrix, forming protrusions of cells with mesenchymal morphology. TOs, therefore, serve as a valuable system to study EVT invasion *in vitro*.

In this study, we investigated in detail the impact of IFN-α and TNF-α on EVT differentiation and invasion using monolayer TSC cultures, TOs, and first-trimester placental and decidual tissue. Our findings indicate that IFN-α and TNF-α do not interfere with EVT differentiation but significantly suppress EVT invasion into both Matrigel matrices and maternal decidual tissue. For the latter, we co-cultured TOs with decidual explants, in which we observed pronounced trophoblast invasion into the stroma and arteries using high-resolution optical sectioning. Transcriptome analysis on Matrigel-invading EVTs suggested that EMT and other pathways associated with cellular invasion are affected by these cytokines, but direct targets that interfere with these pathways remain to be elucidated. As EVT invasion is crucial for proper spiral artery remodeling and a healthy pregnancy, the direct effect of IFN-α and TNF-α on trophoblasts may contribute to the increased risk for adverse pregnancy outcomes in patients with elevated IFN-α/TNF-α activity.

## Results

### IFN-α and TNF-α do not affect monolayer EVT differentiation

For this study, we made use of a TSC line previously generated from first-trimester placental tissue.^46^ This TSC line, as well as additionally generated TSC lines, express the genes encoding IFN-α receptors (*IFNAR1* and *IFNAR2*) and TNF-α receptors (*TNFRSF1A* and *TNFRSF1B*), in the stem cell state and differentiated EVT state (Figure S1).^47^ *TNF* mRNA was endogenously expressed at very low levels in TSCs, whereas transcripts encoding IFN-α were not detected.

**Figure 1:**
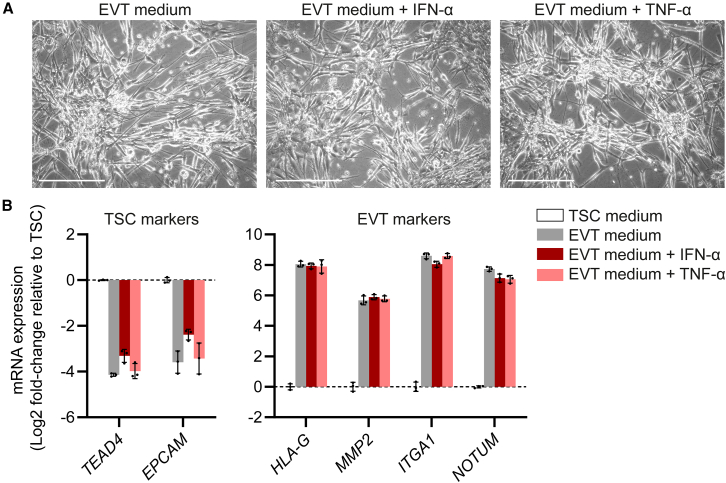
IFN-α and TNF-α do not hinder monolayer EVT differentiation TSCs were induced to undergo monolayer EVT differentiation for 8 days in the presence of IFN-α (5 ng/mL) or TNF-α (10 ng/mL), or without treatment as a control. (A) Representative phase-contrast images at EVT differentiation day 8. Scale bars, 400 μm. (B) TSC and EVT marker mRNA expression measured by RT-qPCR. Bars represent mean log2 fold-change ±SD relative to undifferentiated TSCs (*n* = 3 independent experiments). TSC markers were significantly downregulated and EVT markers significantly upregulated *(p* < 0.01) in EVT conditions relative to the TSC condition (one-way ANOVA with Dunnett’s multiple comparisons test). Differences between treatments were not significant.

To assess the impact of the cytokines on monolayer EVT differentiation, IFN-α or TNF-α was added to the EVT differentiation medium throughout the entire culture period. Irrespective of these treatments, the cells obtained an elongated, mesenchymal morphology typical for EVTs (Figure 1A). Moreover, the cells exhibited clear downregulation of TSC markers and upregulation of EVT markers relative to undifferentiated TSCs (Figure 1B), suggesting that IFN-α and TNF-α do not block EVT differentiation.

### IFN-α and TNF-α inhibit the invasive capacity of EVTs into the Matrigel matrix

Similarly, we added the cytokines to trophoblast organoid medium (TOM), overlaying TSCs seeded in Matrigel, to investigate whether TSCs are able to form organoids under these conditions. Morphology of the organoids seemed normal, and they showed similar mRNA expression of TSC, EVT, and STB markers as untreated organoids (Figures S2A and S2B). Thus, IFN-α and TNF-α did not seem to affect TO formation.

**Figure 2:**
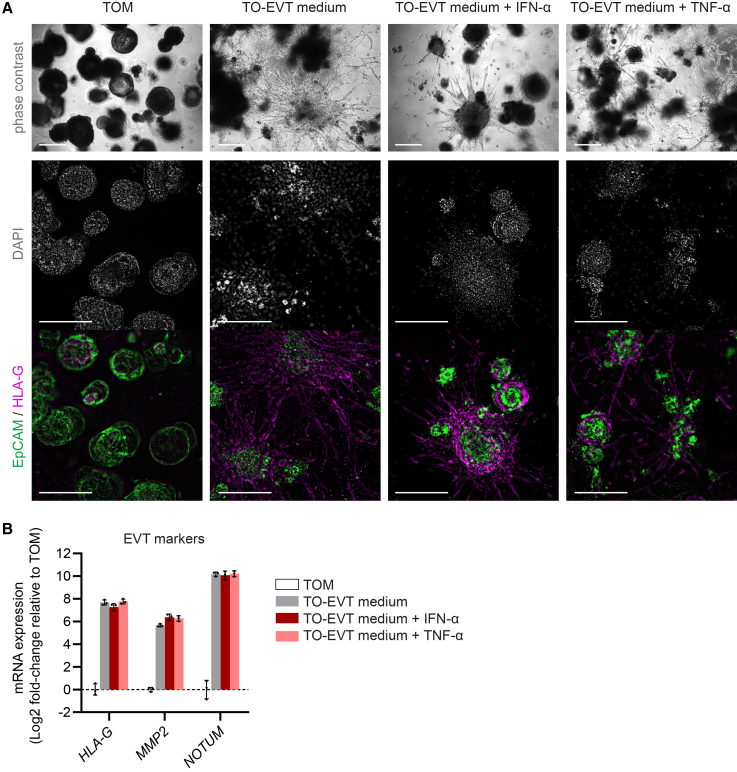
IFN-α and TNF-α inhibit EVT invasion but do not hinder EVT differentiation in trophoblast organoids Organoids were grown in Matrigel domes in trophoblast organoid medium (TOM) for 5 days, and consecutively induced to undergo EVT differentiation in trophoblast organoid (TO)-EVT medium in the presence or absence of IFN-α or TNF-α for 14 days, or retained in unsupplemented TOM. (A) Representative phase-contrast images and immunofluorescence extended depth of field composite images. Organoids were stained for EpCAM (TSC marker, green), HLA-G (EVT marker, magenta) and DAPI (nuclei, white). Scale bars, 400 μm. Additional immunofluorescence images are shown in Figure S4. (B) EVT marker mRNA expression measured by RT-qPCR. Bars represent mean log2 fold-change ±SD relative to untreated organoids in TOM (*n* = 3 independent experiments). For all EVT conditions, EVT marker expression was significantly increased (*p* < 0.001) compared with the TOM condition (one-way ANOVA with Dunnett’s multiple comparisons test).

However, when adding the cytokines to organoids in TO-EVT medium to investigate the effect on EVT invasion, we observed reduced EVT outgrowth from the organoids into the Matrigel matrix (Figure 2A). This was not attributable to impaired EVT differentiation, as mRNA levels of EVT markers were markedly elevated compared with organoids in TOM, and similar to those of organoids in TO-EVT medium without cytokines (Figure 2B). These results were validated using another TSC line from an independent placental tissue donor (Figure S3). Furthermore, immunostaining for HLA-G and MMP2 confirmed the presence of EVTs; however, these cells remained in closer proximity to the organoid bodies (Figures 2A and S4), indicating a reduced invasion capacity into the surrounding Matrigel matrix in the presence of IFN-α or TNF-α.

**Figure 3:**
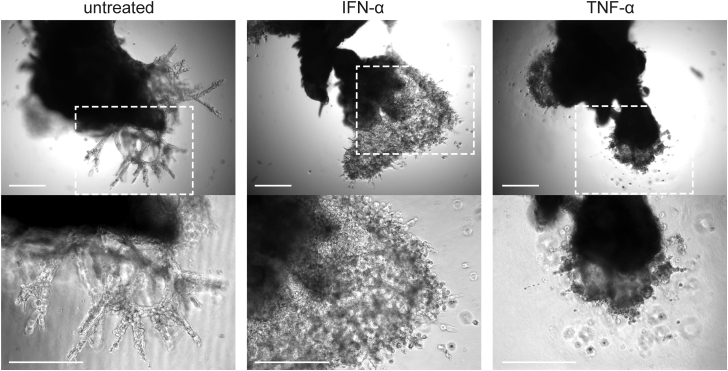
IFN-α and TNF-α affect EVT invasion in first-trimester placental villous explants Representative phase-contrast images of placental villous explants (8 weeks of gestation), which were seeded on Matrigel and treated with IFN-α or TNF-α. Higher magnification images of the indicated areas are shown in the bottom row. Scale bars, 400 μm. Representative images obtained from other tissue donors are shown in Figure S5.

Using placental villous explants, we observed a similar effect: Untreated explants developed prominent outgrowth composed of elongated EVTs, whereas explants treated with IFN-α or TNF-α exhibited altered outgrowth patterns characterized by small, rounded EVTs closely associated with the villous structures (Figures 3 and S5).

**Figure 4:**
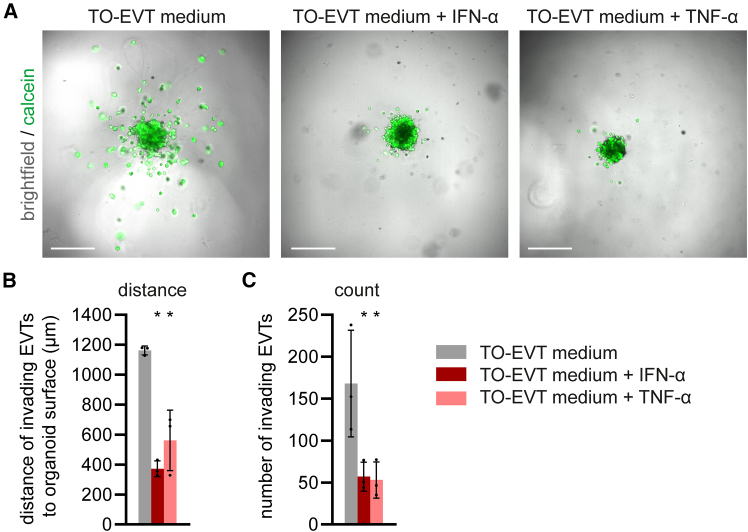
Quantification of EVT invasion using single organoids demonstrates that IFN-α and TNF-α inhibit EVT invasion Trophoblast organoids were transferred individually onto separate wells on top of a layer of Matrigel in TO-EVT medium, with or without IFN-α/TNF-α (10 organoids per condition per experiment). Single organoids were stained with calcein AM, imaged, and analyzed in three dimensions. (A) Representative extended depth of field composite images of Z stacks displays calcein (green) and brightfield. Scale bars, 500 μm. (B) Mean distance of invading EVTs (using the mean of 5 furthest migrated EVTs per image) to the surface of the organoid body ±SD. (C) Mean number of invading EVTs ±SD. Data points represent *n* = 3 independent experiments. ∗*p* < 0.001, compared with untreated control (by generalized linear modeling, with Gaussian distribution for distance measurements and Poisson distribution for count data).

To be able to quantify the invasive capacity of EVTs, we set up a single-organoid system. In this, we induced organoid formation and early EVT differentiation within Matrigel domes for 5 days each, and subsequently transferred the organoids individually onto a Matrigel layer in separate wells, in which they were induced to undergo EVT differentiation for another 9 days. In this system, EVTs invaded the Matrigel as rounded, individual cells, and staining with the live cell marker calcein AM showed that the majority of these cells were alive (Figure 4A). Measuring the distance of invading EVTs to the surface of the organoid body demonstrated that treatment with IFN-α or TNF-α indeed reduced EVT invasion capacity (Figure 4B). Additionally, the number of invasive EVTs, i.e., EVTs detached from the organoid body, was significantly reduced (Figure 4C).

### IFN-α and potentially TNF-α inhibit EVT invasion into the decidua

**Figure 5:**
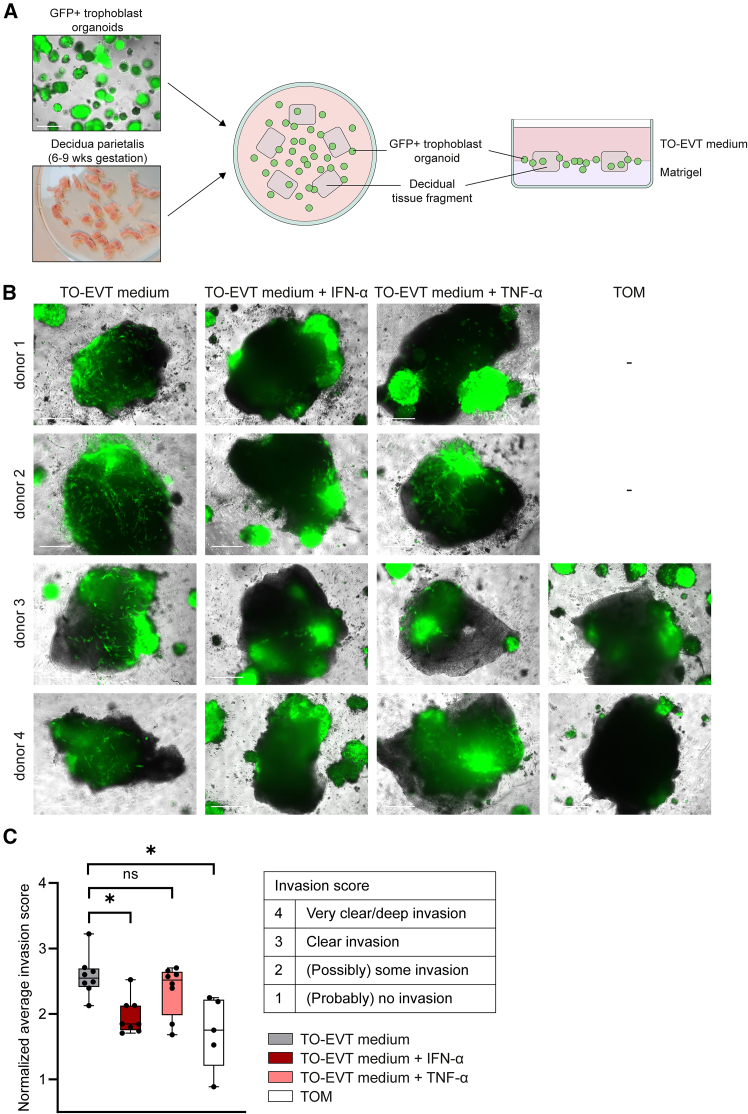
IFN-α and potentially TNF-α inhibit EVT invasion from trophoblast organoids into first-trimester decidual tissue (A) Schematic drawing of the experimental set-up. GFP-positive trophoblast organoids were co-cultured with decidual parietalis tissue fragments (6–9 weeks of gestation) for 8–11 days, with or without IFN-α/TNF-α. (B) Live-cell microscopy images of co-cultures show an overlay of phase contrast and GFP immunofluorescence. Tissue fragments with the greatest extent of organoid invasion out of 4–6 fragments are shown. Scale bars, 500 μm. Representative images obtained from other tissue donors are shown in Figure S6A. (C) Normalized average invasion scores from 4 to 6 tissue fragments. Images used to score the invasion (scoring table) are shown in Figure S6B. Boxplot represents median with interquartile range and min-max whiskers (n = 5–8 independent experiments, using tissue from different donors). ∗*p* < 0.05. ns: not significant (Kruskal-Wallis test with Dunnett’s multiple comparisons test).

To investigate whether IFN-α and TNF-α also disrupt EVT invasion in the context of the maternal decidua, we established a co-culture system of TOs with *ex vivo* first-trimester decidual parietalis tissue. The organoids were generated from TSCs expressing green fluorescent protein (GFP) under the control of a cytomegalovirus (CMV) promoter, resulting in constitutive GFP reporter expression in all cell types. This allowed observation of trophoblast invasion into the decidual tissue. Prior to co-culture, the organoids were cultured for ∼1 week in TO-EVT medium to induce EVT differentiation, with or without the addition of the cytokines. Small fragments of decidua parietalis were transferred onto a layer of Matrigel, and overlayed with the organoids suspended in TO-EVT medium, with or without added cytokines (Figure 5A). Co-cultures were incubated for 8–11 days.

In the untreated EVT condition, we observed clear invasion of GFP-positive TOs into the decidual explants (Figures 5B and S6A). In contrast, the cytokine-treated EVT organoids, particularly the IFN-α-treated and undifferentiated organoids, showed shallower or no invasion, even though these organoids did attach to the decidual tissue. To draw an unbiased conclusion on the effect of the cytokines on the depth of invasion, the images were randomized and manually scored by two independent researchers, blinded to treatment conditions. An ordinal scale between 1 and 4 was used, with a higher value representing deeper invasion, based on a qualitative assessment (Figure S6B). The normalized average invasion score was significantly reduced for the IFN-α-treated and undifferentiated organoids compared with untreated EVT organoids (Figure 5C). Moreover, we noticed that the individual tissue fragments co-cultured with IFN-α-treated or undifferentiated organoids never received the highest invasion score of 4 from both researchers, suggesting that deep invasion is impaired in the presence of IFN-α or in the absence of EVT differentiation. For TNF-α, the normalized average invasion score was slightly lower than for the untreated control, but did not reach statistical significance. Altogether, this co-culture system shows that the cytokines, in particular IFN-α, not only inhibit EVT invasion into Matrigel, but also in decidual tissue, a situation more closely modeling the *in vivo* situation.

### Co-culture of trophoblast organoids with first-trimester decidual tissue shows the invasion of trophoblasts alongside decidual blood vessels

**Figure 6:**
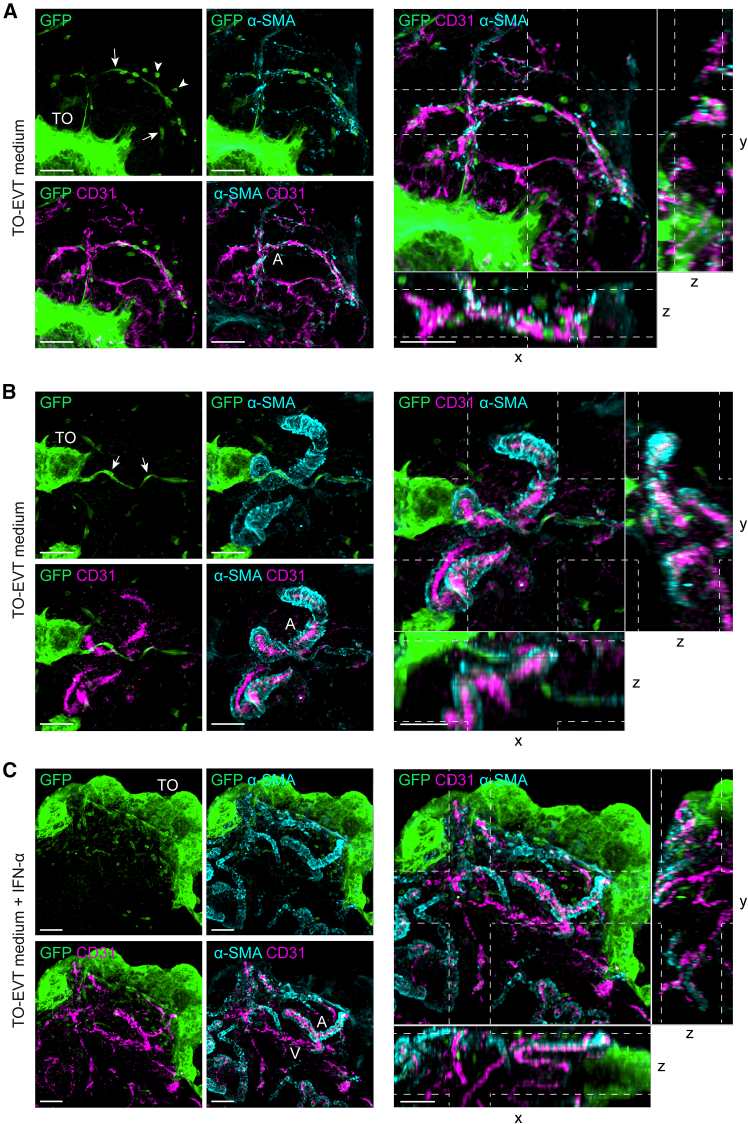
Whole-mount immunostaining of decidua parietalis co-cultured with GFP-positive trophoblast organoids shows invading trophoblasts associating with blood vessels Maximum intensity projection images show GFP (GFP reporter-expressing trophoblasts organoids, green), α-SMA (smooth muscle cell marker, cyan), and CD31 (endothelial cell marker, magenta), representative for *n* = 5 independent tissue donors, with the two fragments with highest extent of EVT invasion for each. (A) Untreated organoid condition, showing organoid-derived trophoblasts in decidual stroma and attached to blood vessels. (B) Additional image of the untreated condition, showing close association of organoid-derived trophoblasts with an artery. (C) IFN-α-treated condition, showing superficial organoids. Scale bars, 100 μm. x, y, z: dimensions of image stacks. TO: trophoblast organoid; arrows: organoid-derived trophoblasts attached to blood vessels; arrowheads: organoid-derived trophoblasts in decidual stroma (in GFP-only images). A: artery; V: vein (in α-SMA + CD31 overlays). See Figure S7 for overview images of fragments for better contextualization.

To better visualize organoid-derived trophoblast invasion into decidual tissue and investigate potential interactions with blood vessels, co-cultures were fixed and subjected to whole-mount immunostaining using antibodies against GFP, the endothelial cell marker cluster of differentiation 31 (CD31; encoded by *PECAM1*), and the smooth muscle cell marker alpha smooth muscle actin (α-SMA; encoded by *ACTA2*). Many tissue fragments contained clearly identifiable spiral arteries, positive for CD31 and α-SMA (Figures 6 and S7). Organoid-derived, GFP-positive trophoblasts were observed invading the decidual stroma and closely associated with arteries (Figures 6A and 6B). Within the stroma, trophoblasts displayed a rounded morphology (arrowheads in Figure 6A, GFP-only image), whereas they adopted a more elongated morphology when in contact with blood vessels (arrows in Figures 6A and 6B, GFP-only image). In the IFN-α-treated condition, organoids remained superficial, not infiltrating the tissue deeply (Figure 6C). Moreover, we did not find clear associations between organoid-derived trophoblasts and blood vessels, as observed in the untreated condition.

### Mechanism(s) underlying reduced EVT invasion

**Figure 7:**
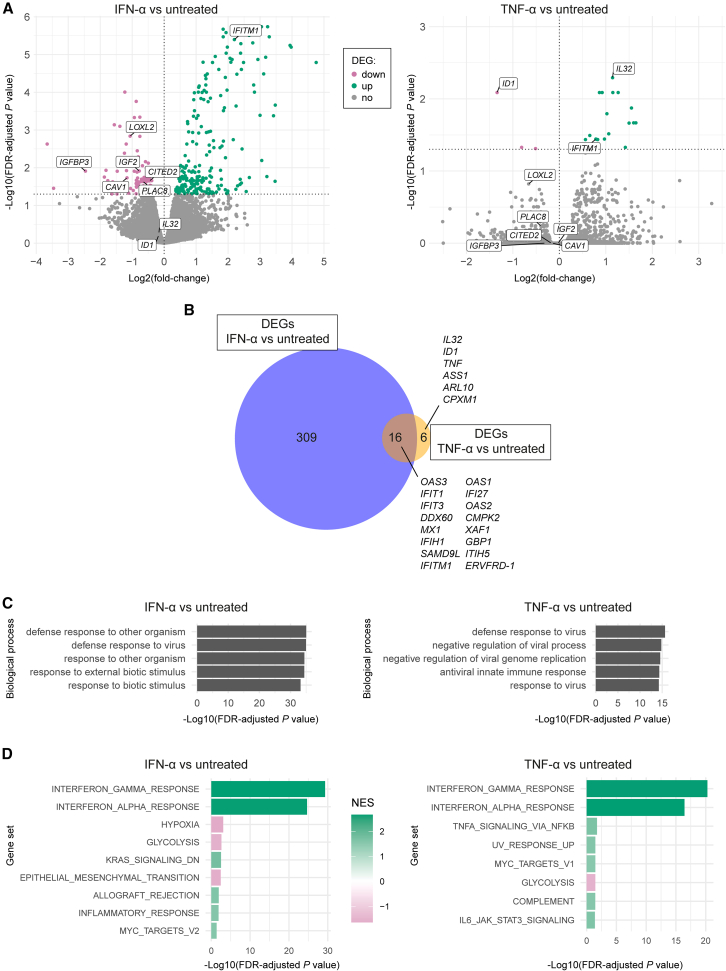
Transcriptome sequencing of EVTs isolated from IFN-α- and TNF-α-treated trophoblast organoids reveals potential mechanisms underlying reduced EVT invasion Organoids were induced to undergo EVT differentiation for 14 days, with or without IFN-α/TNF-α, and used to isolate HLA-G-positive cells (*n* = 3 independent experiments). (A) Volcano plots show differentially expressed genes (DEGs) (FDR-adjusted *p* < 0.05) in IFN-α-treated (left) and TNF-α-treated (right) EVTs compared with untreated control EVTs. Genes mentioned in the text are indicated. (B) Venn diagram shows the overlap between DEGs for IFN-α and TNF-α versus control. Genes specific for TNF-α are sorted by FDR-adjusted *p* value for TNF-α versus control, and the list of overlapping genes between IFN-α and TNF-α is sorted by FDR-adjusted *p* value for IFN-α versus control. (C) Top 5 significantly overrepresented biological processes GO terms and (D) significantly enriched gene sets within DEGs. NES: normalized enrichment score.

To identify mechanism(s) underlying the reduced EVT invasion capacity, we FACS-isolated HLA-G-positive EVTs from cytokine-treated and untreated organoids after full EVT differentiation (Figure S8A) and performed transcriptome sequencing. Viability and percentage of HLA-G-positive cells were not significantly affected by the cytokine treatments (Figure S8B). Compared with untreated controls, we identified 325 differentially expressed genes (DEGs; FDR-adjusted *p* < 0.05) in IFN-α-treated EVTs and 22 DEGs (FDR-adjusted *p* < 0.05) in TNF-α-treated EVTs (Figure 7A). Of these, 16 DEGs overlapped between the IFN-α and TNF-α conditions (Figure 7B). Complete DEG analysis results are provided in Table S1.

Using a panel of markers indicative of the EVT cell state derived from the literature,^41^^,^^42^^,^^48^^,^^49^ we further characterized the EVT phenotype of the cytokine-treated cells (Figure S9). The vast majority of EVT markers (23 out of 25) were not differentially expressed relative to the untreated control, supporting our conclusion that the IFN-α- and TNF-α-exposed cells represent bona fide differentiated EVTs. However, the expression of the invasive EVT markers *PLAC8* and *IGF2* was significantly reduced in IFN-α-treated EVTs (Figure S9), suggesting that IFN-α inhibits the formation of an invasive EVT subtype. Moreover, as PLAC8 and IGF2 are known to promote EVT invasion and migration,^50^^,^^51^ their downregulation may also contribute to the reduced invasive capacity.

Genes upregulated following exposure to either cytokine included many interferon-stimulated genes, demonstrating a clear response to cytokine treatment. Consistent with this, gene ontology (GO) analysis indicated an overrepresentation of GO terms related to viral response among the DEGs (Figure 7C) and pathway analysis indicated enrichment of interferon response gene sets (Figure 7D). Some interferon-induced genes are also known to inhibit trophoblast invasion, such as *IFITM1*,^52^ which was upregulated in IFN-α- and TNF-α-treated EVTs.

Upon IFN-α treatment, there was also an enrichment in genes associated with EMT, hypoxia, and glycolysis, which are implicated in trophoblast invasion^53^^,^^54^^,^^55^^,^^56^^,^^57^ (Figure 7D; see Table S2 for DEGs within these pathways). Notably, these pathways contained several DEGs that are known to promote trophoblast invasion, such as *IGFBP3*,^58^ *CITED2*,^59^ *LOXL2,*^60^ and *CAV1*,^61^ all of which were downregulated upon IFN-α treatment. Downstream regulation of these pathways and genes may therefore underlie the inhibitory effect of IFN-α on EVT invasion.

Among TNF-α-induced DEGs, the glycolysis gene set was also enriched (Figure 7D), although it did not contain individual DEGs. To further investigate the mechanism underlying TNF-α-mediated reduced invasion, we functionally tested the most prominent DEG in the TNF-α volcano plot (Figure 7A): *IL32*, encoding the cytokine IL-32. As *IL32* was upregulated upon TNF-α treatment, we hypothesized that the addition of IL-32 mimics the effect of TNF-α. However, treatment of organoids with IL-32 (10 ng/mL) promoted EVT invasion instead (Figure S10). Upregulation of *IL32* could therefore not explain the TNF-α-mediated reduced invasiveness. The gene *ID1*, which was downregulated upon TNF-α treatment, is known from the literature to promote trophoblast invasion,^58^ and its downregulation therefore potentially contributes to the reduced invasiveness.

## Discussion

Immune-mediated inflammatory diseases, in particular SLE, are strongly associated with adverse pregnancy outcomes. This is thought to be caused by the disruption of the local immune balance and failure of maternal-fetal tolerance.^62^ However, the direct effects of the associated pro-inflammatory cytokines on trophoblast development remain poorly understood. In this study, we investigated the effects of IFN-α and TNF-α on EVT differentiation and invasion using multiple model systems. Using different platforms such as TSC monolayer, TOs, and first-trimester placental villous explants, we found that IFN-α and TNF-α inhibit EVT invasion into Matrigel, despite normal EVT differentiation. In a co-culture of TOs with first-trimester decidual explants, particularly IFN-α inhibited EVT invasion into decidua in a similar way as within Matrigel. Transcriptome sequencing on the EVT subpopulation from TOs suggested that IFN-α and TNF-α affected several pathways and genes implicated in invasion.

EVT differentiation and invasion into the maternal decidua are essential placentation processes, taking place predominantly during the first trimester.^3^^,^^7^ Within the decidua, EVTs remodel spiral arteries into large-diameter vessels with reduced contractility, which enables proper maternal blood flow to the intervillous space. Incomplete EVT invasion and spiral artery remodeling can lead to discontinuous and high-rate blood flow, causing oxidative stress, damage to the placental villous tree, and disturbed maternal-fetal exchange, as seen in early-onset preeclampsia.^63^ As our results indicate that IFN-α and TNF-α directly inhibit EVT invasion, this mechanism may contribute to the increased incidence of preeclampsia and other adverse outcomes in SLE pregnancies.

To be able to thoroughly study EVT invasion in a physiological way, we used two assays: a single-organoid system and a co-culture system of GFP-expressing TOs with first-trimester decidual explants. The single-organoid assay allowed the quantification of EVT invasion into the extracellular matrix, while the co-culture assay enabled qualitative assessment of EVT interaction with decidual tissue. GFP reporter-expressing TSCs allowed the visualization of EVTs within different decidual compartments, such as stroma, blood vessels, and glands, using whole-mount immunostaining. The presence of these structures and the three-dimensionality make this a more physiological and translational model of EVT invasion than, for example, the widely used transwell invasion assays, and other *in vitro* culture systems out of their tissue context. Moreover, since TSCs can be genetically manipulated, both assays offer excellent opportunities to study the role of specific genes in EVT invasion and early placenta formation.

Within the decidual tissue, we observed organoid-derived trophoblasts in the stroma and in close contact with blood vessels. This suggests that EVTs are attracted by various decidual cell types in this system, similar to *in vivo*.^64^ This observation also highlights the potential of this system for studying spiral artery remodeling, although the currently maximal time window of the co-culture might be a limiting factor. Spiral artery remodeling takes place between 6 and 20 weeks of gestation, with individual arteries undergoing remodeling asynchronously, but the duration for individual arteries is unknown.^6^ This would be worth further investigation with further optimization of the co-culture system and high-resolution imaging. Additional characterization would also allow studying EVT association with, for example, decidual immune cells, glandular epithelium, and lymphatics. Moreover, spatial omics techniques could be used to determine whether organoid-derived EVT localization correlates with altered gene expression, and how this compares to *in vivo* profiles of EVT subtypes as determined by others.^49^

An important question is whether the cytokine concentrations used here reflect those in patients. In general, cytokines primarily function via paracrine and autocrine signaling to regulate local immune responses, and systemic levels are therefore low.^65^ In SLE and other immune-mediated inflammatory diseases, however, systemic levels are elevated, especially during disease flares.^66^^,^^67^ Local cytokine concentrations are probably more relevant for EVT invasion, but they are difficult to measure. For the choice of concentrations, we relied on concentrations used in the literature. In the co-culture system, decidual cells are also exposed to the added cytokines, and most likely consume them,^68^ possibly interfering with results. This may explain the weaker effect of TNF-α in co-culture with decidua than in culture of organoids alone. Another explanation would be that the co-culture system may not be sensitive enough to detect subtle differences, as many factors are involved.

To identify the molecular mechanisms by which IFN-α/TNF-α reduce EVT invasion, we performed transcriptome sequencing on HLA-G-positive EVTs isolated from cytokine-treated organoids. Pathway analysis revealed enrichment of genes associated with EMT, hypoxia, and glycolysis, pathways known to be involved in trophoblast invasion.^53^^,^^54^^,^^55^^,^^56^^,^^57^ In addition, several genes that promote trophoblast invasion were downregulated, including *PLAC8*, *IGF2*, *IGFBP3*, *CITED2*, *LOXL2,* and *CAV1* upon IFN-α treatment, and *ID1* upon TNF-α treatment. Dysregulations of these pathways and genes induced by IFN-α/TNF-α may underlie the observed reduction in EVT invasion. As several DEGs overlapped between IFN-α and TNF-α treatment, including the invasion-related interferon-response gene *IFITM1*, the underlying mechanisms may be partially shared.

IL-32, which was upregulated following TNF-α treatment, had the opposite effect of TNF-α and promoted EVT invasion, in line with findings of a study using HTR-8/SVneo cells.^69^ The TNF-α-induced upregulation of *IL32* may therefore reflect a compensatory mechanism. In addition, IL-32 has been reported to upregulate *TNF*,^70^ suggesting that these cytokines may form a negative feedback loop regulating EVT invasion.

Simoni et al. reported that IFN-β, another IFN, inhibits EVT invasion in an organ-on-a-chip model using primary EVTs and endometrial endothelial cells.^28^ Consistent with our findings, they showed that IFN-β reduces EVT invasion through Matrigel toward endothelial cells, accompanied by limited emergence of invasive EVTs and alterations in EMT-related gene expression. However, the study did not distinguish between direct and indirect effects on trophoblasts, as both endothelial cells and EVTs were exposed to IFN-β. Moreover, the absence of other decidual cells, such as stromal and immune cells, precluded investigation of interactions with additional components of the microenvironment. Our study, using TOs and co-cultures with decidual tissue, therefore provides a valuable follow-up. The consistency of the findings further supports a role for IFNs in regulating EVT invasion.

In pregnancy, type I IFNs and TNF-α at the maternal-fetal interface are known to play a role in protecting the fetus against bacterial and viral infection.^26^ Considering their effect on EVT invasion, secretion of low levels of these cytokines by decidual cells might play an additional role in preventing excessive invasion into the uterine wall. Together with other signals, they may balance invasion depth. Interestingly, when interstitial EVTs undergo terminal differentiation into placental bed giant cells *in vivo*, they upregulate type I IFN receptors.^49^ As the formation of giant cells is thought to be a mechanism preventing excessive invasion,^71^ it is conceivable that type I IFN signaling plays a role in that. It would be interesting to further investigate the involvement of these cytokines in conditions such as placenta accreta, where the placental villi and EVTs invade the myometrium.^72^ Beyond immune-mediated inflammatory diseases, the effect of these cytokines on EVT invasion may also contribute to the increased risk for adverse pregnancy outcomes upon infection and in other chronic inflammatory conditions such as obesity.^73^^,^^74^^,^^75^

Women with active or recently diagnosed SLE are advised to postpone pregnancy until disease activity is low or in remission.^76^ A deeper understanding of the mechanisms driving pregnancy complications in SLE and other immune-mediated inflammatory diseases may provide a rationale for such management as well as for medical treatment decisions. Moreover, it may reveal new targets for preventative treatments. This work improves our understanding of early placenta development and offers mechanistic insights into the increased risk for adverse pregnancy outcomes in patients with SLE.

### Limitations of the study

Limitations of the co-culture system combining TOs with decidual tissue that we experienced include dependency on donor tissue availability, occasional poor tissue quality, and technical complexity. In addition, high inter-donor and inter-experimental variability in terms of invasion depth required multiple repetitions of the experiment and normalization. Nevertheless, the co-culture system opens up many possibilities for future research.

Due to the time point of sampling for transcriptome sequencing, DEGs found in cytokine-treated organoid-derived EVTs may reflect secondary, downstream changes rather than primary, early cytokine-driven responses. Sampling at an earlier time point after cytokine exposure may provide more information about direct targets, but this was not practically feasible, as it takes several days before invasive EVTs arise in sufficient numbers for purification and subsequent RNA sequencing.

## Resource availability

### Lead contact

Requests for further information and resources should be directed to and will be fulfilled by the lead contact, Gijs B. Afink (g.b.afink@amsterdamumc.nl).

### Materials availability

This study did not generate new unique reagents or devices.

### Data and code availability

Transcriptome sequencing data have been deposited in the Gene Expression Omnibus database under accession number GSE302798 and are publicly available as of the date of publication. This paper does not report original code. Any additional information required to reanalyze the data reported in this paper is available from the lead contact upon request.

## Acknowledgments

We would like to thank the donors for the placental and decidual tissue that made this study possible. We would also like to thank present and past lab members of the Chuva de Sousa Lopes and Afink groups for fruitful discussions and technical advice. This work made use of the Dutch National E-Infrastructure with support of the SURF Cooperative, using grant no. EINF-14364. This work was supported by the 10.13039/501100009708Novo Nordisk Foundation (NNF21CC0073729, reNEW to SMCSL).

## Author contributions

A.J.v.V. and G.B.A. designed research; A.J.v.V., F.L., S.B., R.K., L.B., and B.H. performed experiments; A.J.v.V. and R.K. analyzed data; A.M.M.v.P., W.D., S.M.C.d.S.L., and G.B.A. critically evaluated research; A.J.v.V. and G.B.A. wrote the paper.

## Declaration of interests

The authors declare no competing interests.

## Declaration of generative AI and AI-assisted technologies in the writing process

During the preparation of this work, the authors used ChatGPT (OpenAI) in order to rephrase pieces of text to improve readability. After using this tool, the authors reviewed and edited the content as needed and take full responsibility for the content of the published article.

## STAR★Methods

### Key resources table

**Table:** 

REAGENT or RESOURCE	SOURCE	IDENTIFIER
**Antibodies**		

FITC-conjugated anti-HLA-G antibody	EXBIO	Cat#1F-292-C100; RRID:AB_10735947
FITC-conjugated mouse IgG1 isotype control	BD	Cat#555748; RRID:AB_396090
Antibodies for immunofluorescence, see Table S4	N/A	N/A

**Biological samples**		

First-trimester human placental tissue	Human Immune System Mouse Facility, Amsterdam University Medical Center	N/A
Human first-trimester human decidua parietalis tissue	Leiden University Medical Center	N/A

**Chemicals, peptides, and recombinant proteins**		

Matrigel growth-factor reduced basement membrane matrix	Corning	Cat#356231
Advanced DMEM/F-12	Life Technologies	Cat#12634010
Penicillin-Streptomycin	Gibco	Cat#15140122
N-acetyl-L-cysteine	Sigma-Aldrich	Cat#A9165
B27 supplement minus vitamin A	Gibco	Cat#12587010
L-glutamine	Life Technologies	Cat#25030-024
N2 supplement	Gibco	Cat#17502048
Y-27632	STEMCELL Technologies	Cat#72304
PGE2	Tocris Bioscience	Cat#2296
A83-01	STEMCELL Technologies	Cat#72022
CHIR99021	STEMCELL Technologies	Cat#72052
Human recombinant HGF	PeproTech	Cat#100-39
Human recombinant R-spondin 1	R&D Systems Bio-Techne	Cat#4645-RS-025
Human recombinant FGF2	PeproTech	Cat#100-18C
Human recombinant EGF	STEMCELL Technologies	Cat#78136
2-mercaptoethanol	Gibco	Cat#31350010
BSA	Sigma-Aldrich	Cat#A9205
ITS-X supplement	Gibco	Cat#51500056
Heregulin-beta 1	STEMCELL Technologies	Cat#78071
KnockOut Serum Replacement	Gibco	Cat#10828010
DMEM/F-12 + GlutaMAX	Gibco	Cat#31331-028
Human recombinant TNF-α	PreproTech	Cat#AF-300-01A
Human recombinant IFN-α 2A	STEMCELL Technologies	Cat#78076
Human recombinant IL-32β	R&D Systems	Cat#6769-IL-025
Dispase II	Sigma-Aldrich	Cat#D4693
Bright Diluent	Immunologic	VWRKUD09
DAPI	Sigma-Aldrich	Cat#D9542
Cell Recovery Solution	Corning	Cat#354253
Calcein AM	eBioscience	Cat#650853
Cell Banker I	AMSBIO	Cat#11910
Gentamicin	Gibco	Cat#15710-049
Saponin	Acros Organics	Cat#419231000
Ethyl cinnamate	Sigma-Aldrich	Cat#W243000
TrypLE	Gibco	Cat#12604
Bioscience Fixable Viability Dye eFluor 780	Invitrogen	Cat#65-0865-14

**Critical commercial assays**		

KAPA mRNA HyperPrep kit	Roche	Cat#08098123702

**Deposited data**		

Transcriptome sequencing data	This paper	GEO: GSE302798
Transcriptome sequencing data	Van Voorden et al.^47^	GEO: GSE282356

**Experimental models: Cell lines**		

Human trophoblast stem cells	Van Voorden et al.^46^	TSC_1; TSC_3

**Oligonucleotides**		

Primers for RT-qPCR, see Table S3	N/A	N/A

**Recombinant DNA**		

MISSION® pLKO.1-puro-CMV-TurboGFP Positive Control Plasmid	Merck	SHC003

**Software and algorithms**		

Leica Application Suite X (v3)	Leica Microsystems	N/A
Imaris (v10.1.1)	Oxford Instruments-Andor Technology	N/A
ImarisViewer (v10.2.0)	Oxford Instruments-Andor Technology	N/A
FlowJo (v10)	BD Biosciences	N/A
GraphPad Prism (v10.2.0)	GraphPad Software	N/A
R (v4.4.1)	R Core Team	N/A

**Other**		

Nunc™ cell-culture treated 4-well plate	Thermo Scientific	Cat#176740
Nunc™ 96-well optical-bottom microplate, black, TC surface	Thermo Scientific	Cat#10281092

### Experimental model details and study participant details

#### Human tissue collection

First-trimester placental and decidual tissue were obtained from the Human Immune System Mouse Facility of the Amsterdam University Medical Center, Amsterdam, and de-identified prior to use in the current study. The collection of extra-embryonic tissue was approved by the Amsterdam University Medical Center Biobank Review Committee (AMC 2016_246). Alternatively, decidual tissue was collected from the Leiden University Medical Center, as approved by the Medical Ethical Committee of the Leiden University Medical Center (B21.054). The human tissue was donated for scientific research with written informed consent from donors undergoing elective abortion without medical indication.

#### TSC and organoid culture and differentiation

Culture and differentiation of TSCs and trophoblast organoids were performed as described in our previous work.^46^ In short, human male TSC lines, previously isolated from first-trimester placental tissue,^46^ were cultured and induced to undergo EVT differentiation in monolayer following protocols of Okae et al*.*^41^ Trophoblast organoids were generated by seeding TSCs into domes of Matrigel growth-factor reduced basement membrane matrix (Corning, 356231). According to protocols established by others,^42^^,^^43^ organoids were cultured in trophoblast organoid medium (TOM) (Advanced DMEM/F-12 with 0.5% Penicillin-Streptomycin, 1.25 mM N-acetyl-L-cysteine, 1X B27 supplement minus vitamin A, 2 mM L-glutamine, 1X N2 supplement, 2 μM Y-27632, 2.5 μM PGE2, 0.5 μM A83-01, 1.5 μM CHIR99021, 50 ng/mL human recombinant HGF, 80 ng/mL human recombinant R-spondin 1, 100 ng/mL human recombinant FGF2 and 50 ng/mL human recombinant EGF) (See key resources table for details of chemicals used in organoid culture media), and induced to undergo EVT differentiation in (TO-)EVT medium A (Advanced DMEM/F-12 with 0.1 mM 2-mercaptoethanol, 0.5% Penicillin-Streptomycin, 0.3% BSA, 1% ITS-X supplement, 100 ng/mL heregulin-beta 1, 7.5 μM A83-01, 4% KnockOut Serum Replacement) typically for 7 days, followed by TO-EVT medium B (medium A without heregulin-beta 1 for another 7 days. TSCs and organoids were kept at 37 °C in 5% CO2 and medium was refreshed every 2–3 days.

#### Placental villous explant culture

First-trimester placental tissue was washed in PBS. Placental villi were dissected and seeded into the center of wells of a 48-wells plate pre-coated with 50 μL of Matrigel, after which they were spread using a needle (5 fragments per condition). To allow attachment to the Matrigel, the explants were overlaid with 20 μL DMEM/F-12 + GlutaMAX (Gibco, 31331-028) with 1% penicillin-streptomycin, and incubated at 37 °C in 5% CO_2_ and 5% O_2_ for 4 h or overnight. Subsequently, 250 μL of the same medium including treatment was added, and the explants were incubated for 5–7 days, with medium refreshments every 2–3 days.

### Method details

#### Cytokine treatment

Human recombinant TNF-α (PreproTech, AF-300-01A) or IFN-α 2A (STEMCELL Technologies, 78076) were added to the medium in a concentration of 10 ng/mL and 5 ng/mL, respectively. Human recombinant IL-32β (R&D Systems, 6769-IL-025) was added in a concentration of 10 ng/mL. These concentrations were based on concentrations used in the literature, and were not toxic to our TSC lines.

#### Lentiviral transduction

To generate green fluorescent protein(GFP)-expressing TSCs, 5x10^4^ cells were transduced with lentivirus 24 h after seeding. The lentivirus was produced in-house using standard packaging protocols with the MISSION pLKO.1-puro-CMV-TurboGFP Positive Control Plasmid (Merck, SHC003). Forty-eight hours post-transduction, cells with high GFP fluorescence were isolated using a Sony SH800 cell sorter, and maintained as TSC monolayer culture.

#### RNA isolation and RT-qPCR

RNA isolation, cDNA synthesis and RT-qPCR of TSCs and organoids were performed as described in our previous work.^46^ To release organoids from the Matrigel, the domes were incubated with 2 mg/mL dispase II (Sigma-Aldrich, D4693), for 2 × 5 min at 37 °C, with resuspending in between incubations using a 1% BSA-coated tip. The organoids were then washed in 1 mM EDTA/PBS, and subsequently used for lysis. Primer sequences used for RT-qPCR are provided in Table S3.

#### Immunofluorescence imaging of organoids

For immunofluorescence imaging, organoid domes were fixed in 2% PFA/PBS for 30 min at 4 °C, and immunostained within the remaining Matrigel. Permeabilization and blocking was done in 0.5% Triton X-100 + 4% BSA in PBS for at least 4 h or overnight at 4 °C. Primary antibodies as well as secondary antibodies with 2 μg/mL DAPI (Sigma-Aldrich, D9542) were diluted in Bright Diluent (Immunologic, VWRKUD09), and both stainings were executed for 2 nights at 4 °C. See Table S4 for antibody dilutions and product information. Washings were done in 0.1% Triton X-100 + 0.2% BSA in PBS for 3 × 30 min at room temperature. Organoids were cleared in 50% glycerol/dH2O + 2.5 M fructose, and imaged on a Leica Thunder Imager DMi8 inverted wide-field microscope. To enhance contrast, grayscale values were adjusted in Leica Application Suite X (Leica Microsystems, v3). All settings and adjustments were kept equal between conditions.

#### Single-organoid EVT invasion assay

To quantify EVT invasion from individual organoids, organoids were generated within Matrigel domes and cultured for 5 days in TOM and 5 days in TO-EVT medium A. Subsequently, organoids were released from the Matrigel domes by incubation in Cell Recovery Solution (Corning, 354253) for 1 h on ice. Using a stereo microscope and 0.1% BSA-coated pipette tips, single organoids were then transferred into the center of wells of a 96-wells plate pre-coated with 40 μL Matrigel (10 organoids per condition per independent experiment). The organoids were overlaid with TO-EVT medium A, in which they were cultured for 2 extra days, and then switched to TO-EVT medium B for another 7 days. Incubation was done at 37 °C in 5% CO_2_, and medium was refreshed every 2–3 days. Organoids were stained with 4 μM Calcein AM (eBioscience, 650853) for 30 min at 37°C. z stack images (10 μm steps) were taken of the single organoids, including all invading EVTs, using a Thunder Imager DMi8 wide-field fluorescence microscope (Leica Microsystems), and analyzed using Imaris software (Oxford Instruments-Andor Technology, v10.1.1).

#### Trophoblast organoid–decidua co-culture

Decidua parietalis tissue (6–9 weeks gestation) was washed in PBS and dissected into ∼1–3 mm cubes under a stereomicroscope. These fragments were either immediately used, or stored in Cell Banker I (AMSBIO, 11910) in liquid nitrogen and thawed on the starting day of co-culture.

GFP-transduced TSCs were used to generate organoids in Matrigel domes for 5 days in TOM and induced to undergo EVT differentiation for 7–9 days. Organoids were then released from the Matrigel using Cell Recovery Solution for 20 min on ice, and resuspended in TO-EVT medium B with 50 μg/mL gentamicin (Gibco, 15710-049). The decidual tissue fragments were placed onto a layer of 200 μL Matrigel in wells of a Nunc cell-culture treated 4-wells plate (Thermo Scientific, 176740) (4–6 fragments per well), if possible with the luminal epithelial side facing upwards, and evenly distributed using a needle. The plate was incubated for 25 min at 37 °C in 5% CO_2_ and 5% O_2_ to allow the Matrigel to set, followed by another 20 min upside-down. Next, the organoid suspension was added on top of the Matrigel layer. Alternatively, DMEM/F12 + GlutaMAX with 1% pen/strep and 50 μg/mL gentamicin was first added, and replaced by the organoid suspension in TO-EVT medium B later that day. The medium was refreshed every 2–3 days and the co-culture was analyzed after 8–11 days.

#### Immunofluorescence imaging of decidual tissue

Co-cultures were washed twice with PBS, after which decidual fragments were transferred to individual wells of a 48-wells plate using tweezers. They were fixed and permeabilized in 4% PFA/PBS with 1% Triton X-100 for 1 h at room temperature, after which they were washed 3 × 20 min in PBS at room temperature and stored at 4 °C until further processing.

For whole-mount immunostaining in a 48-wells plate, non-specific binding sites of the tissue fragments were first blocked by incubation in 1% BSA/PBS with 1% Triton X-100 and 0.1% saponin (Acros Organics, 419231000) (blocking/permeabilization buffer) overnight at 4 °C. The next day, primary antibodies, diluted in blocking/permeabilization buffer, were added to the tissue fragments to incubate 3 nights at 4 °C on a shaking platform. See Table S4 for antibody dilutions. Secondary antibody incubation (optionally in the presence of 2 μg/mL DAPI) was done for 2 nights under the same conditions. Washing was done in between and after incubation steps in PBS with 0.2% Triton X-100 for 6 × 30 min at room temperature. Subsequently, tissue fragments were transferred to 1.5 mL Eppendorf tubes and incubated 2 × 30 min in 100% ethanol. Next, they were cleared in ethyl cinnamate (Sigma-Aldrich, W243000) for 2 days on a shaking platform, and kept at room temperature.

Using a micro spatula, the cleared samples were transferred to a Nunc 96-well optical-bottom black microplate (Thermo Scientific, 10281092) with 150 μL ethyl cinnamate per well. Whole samples were imaged on an Andor Dragonfly 500 spinning disk confocal microscope with 3–6 μm step size. Images were adjusted using ImarisViewer software (Oxford Instruments-Andor Technology, v10.2.0).

#### Isolation of EVTs from organoids

To be able to perform RNA sequencing on the EVT fraction of the EVT-differentiated organoids, we isolated the HLA-G-positive cells. Organoids were induced to undergo EVT differentiation for 14 days, after which they were released from the Matrigel domes using dispase II as described above. To make the organoids single cell, they were incubated in TrypLE (Gibco, 12604) for 5 min at 37 °C, and filtered through a 70 μm filter. The flow-through cells were stained with FITC-conjugated anti-HLA-G antibody (EXBIO, 1F-292-C100, 1:200) or FITC-conjugated mouse IgG1 isotype control (BD, 555748, 1:200) in PBS supplemented with 0.05% BSA and 0.01% NaN_3_ for 15 min at room temperature, and with Bioscience Fixable Viability Dye eFluor 780 (Invitrogen, 65-0865-14) in PBS for another 15 min. The HLA-G-positive cell fraction was identified based on the isotype control and collected using a FACSAria Ilu SORP flow cytometric cell sorter (Becton Dickinson). Doublets and dead cells were excluded, and data were analyzed using FlowJo software (BD Biosciences, v10). The isolated cells were lysed and used for RNA isolation.

#### Transcriptome sequencing

Total RNA libraries were prepared using the KAPA mRNA HyperPrep kit (Roche, 08098123702), and 150 bp paired-end sequencing was performed on an Illumina NovaSeq X Plus system. Quality control, mapping and data analysis were performed as previously described,^46^ with the following alterations in version numbers: Ensembl (v107), RSeQC (v5.0.1), MultiQC (v1.13), R programming language (v4.4.1), EdgeR Bioconductor package (v4.2.2), sva package (3.54.0). Gene set enrichment analysis was performed using the fgsea Bioconductor package (v1.32.4), and Venn diagrams were created using the VennDiagram package (1.7.3).

### Quantification and statistical analysis

Statistical analyses were performed in GraphPad Prism (GraphPad Software, v10.2.0) or R programming language (v4.4.1). *p* < 0.05 was considered statistically significant. All statistical details of experiments can be found in the figure legends.

## Supplemental information

Document S1. Figures S1–S10, Tables S2–S4, and supplemental references

Table S1. Results from DGE analysis in EVT fraction of IFN-α/TNF-α-treated organoids compared with untreated controls

Data S1. Raw Data-Fig2A_IFNA_DAPI_RawImageFile
