# Supplementary material for: The pro-inflammatory cytokines IFN-α and TNF-α inhibit organoid-derived extravillous trophoblast invasion
Source: iScience. 2026 Jun 20;29(7):116431. doi: 10.1016/j.isci.2026.116431 (PMC13315761; doi:10.1016/j.isci.2026.116431)
Supplement: Document S1. Figures S1–S10, Tables S2–S4, and supplemental references [file mmc1.pdf]

iScience, Volume *29*

Supplemental information

The pro-inflammatory cytokines IFN-α and TNF-α inhibit organoid-derived extravillous trophoblast invasion

A. Jantine van Voorden, Fangxu Lin, Souad Boussata, Remco Keijser, Liana Barenbrug, Bente Horselenberg, Ans M.M. van Pelt, Wendy Dankers, Susana M. Chuva de Sousa Lopes, and Gijs B. Afink

SUPPLEMENTARY FIGURES

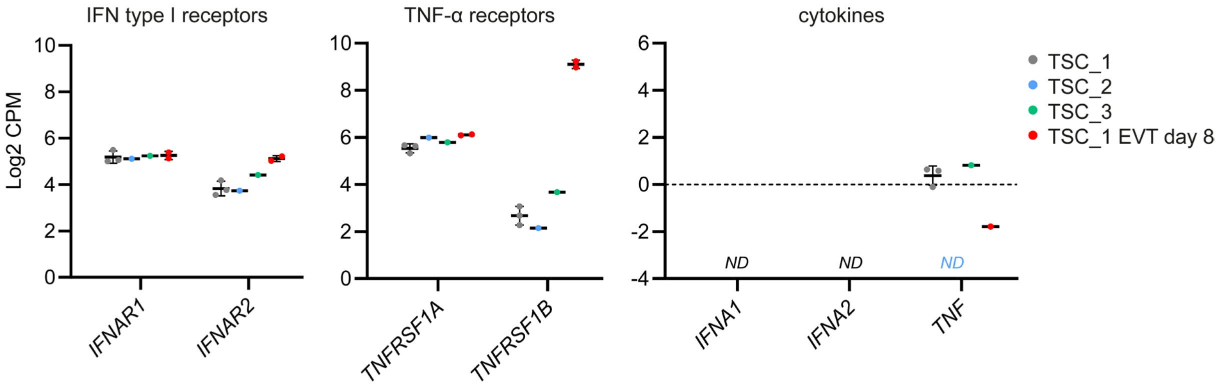

Fig. S1. TSC lines express IFN type I receptors, TNF-a receptors, low levels of *TNF* but no *IFNA*, under proliferative and EVT-differentiation conditions. Data points represent batch-corrected Log2 counts per million (CPM) in previously generated transcriptome data[1] of three previously generated TSC lines (n=1-3 experimental replicates)[2]. TSC_1 was used in the present study. ND: not detectable.

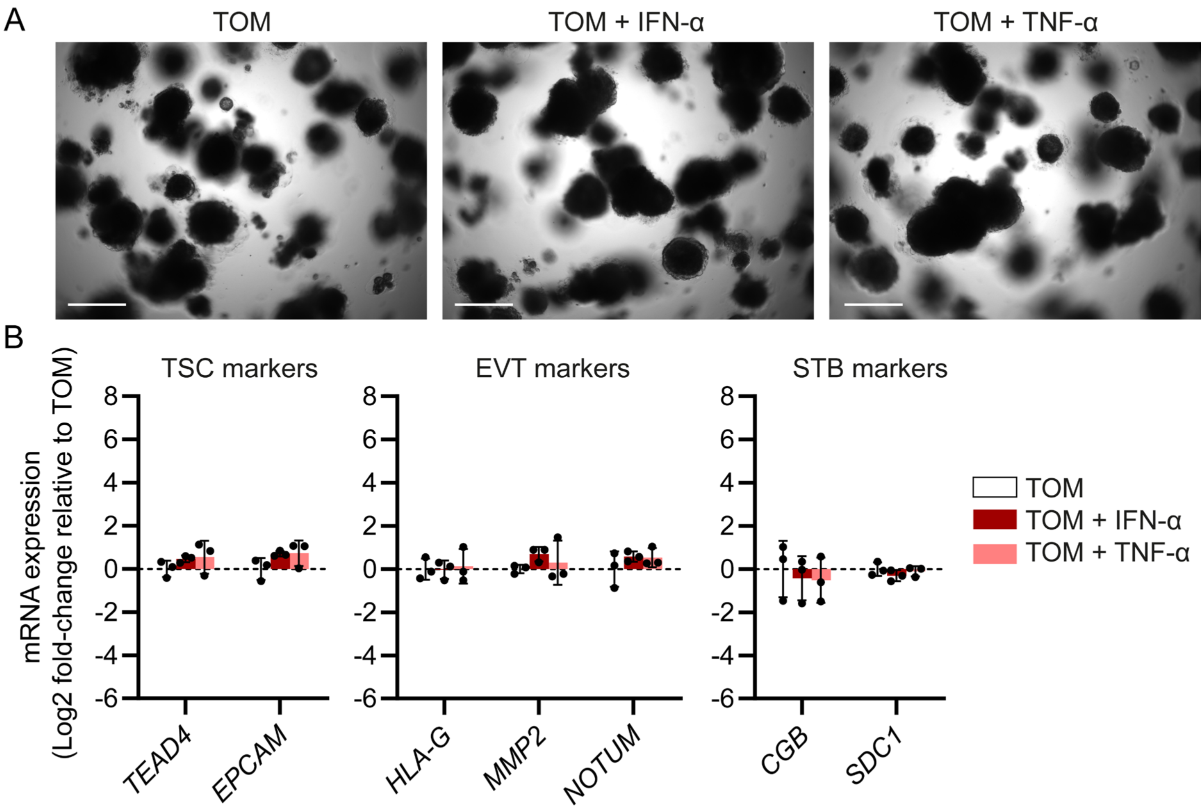

Fig. S2. IFN-α and TNF-α do not affect trophoblast organoid formation. Organoids were grown in Matrigel domes in trophoblast organoid medium (TOM) for 5 days, followed by another 14 days in the presence or absence of IFN-α (5 ng/μl) or TNF-α (10 ng/μl). (A) Representative phase-contrast images. Scale bars: 400 μm. (B) Expression of TSC, EVT and STB markers on the mRNA level, measured by RT-qPCR. Bars represent mean Log2 fold-change ± SD relative to the mean of untreated organoids (n=3 independent experiments). All results were non-significant compared with the untreated TOM condition (one-way ANOVA with Dunnett's multiple comparisons test).

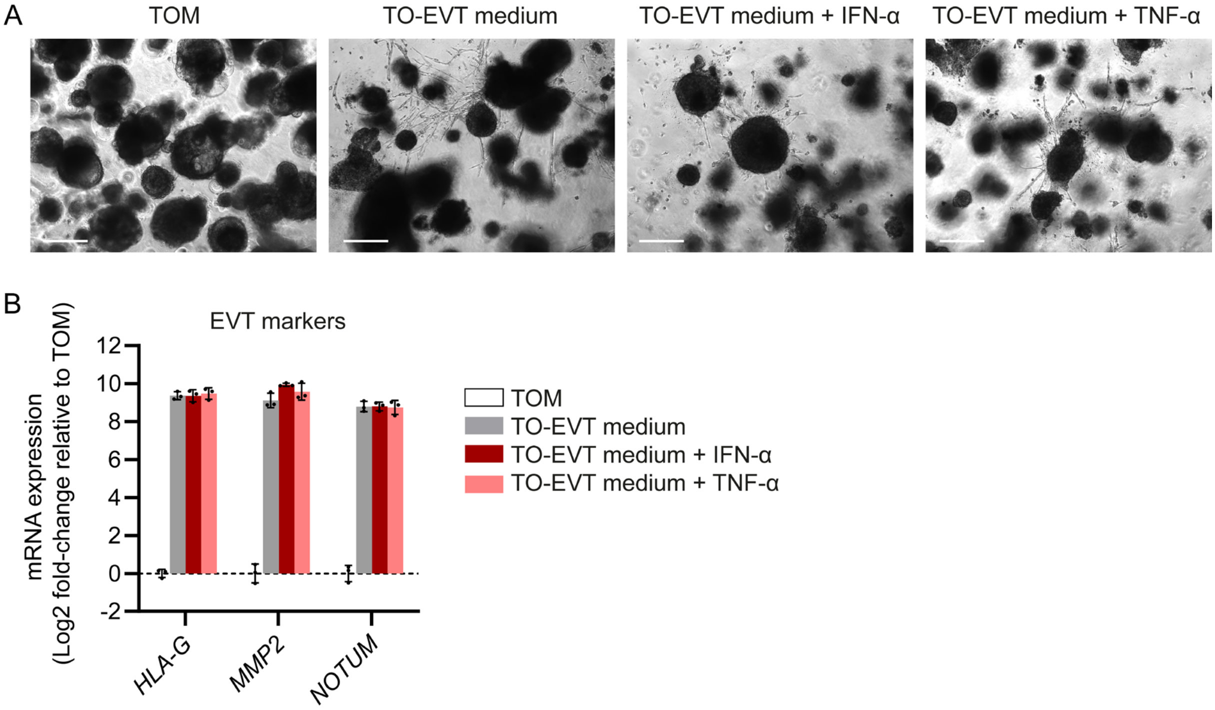

Fig. S3 (related to Fig. 2). Validation of organoid EVT differentiation and invasion experiment with/without IFN-α and TNF-α using another TSC line. Organoids were generated from the TSC_3 line[2] in Matrigel domes for 5 days and induced to undergo EVT differentiation in TO-EVT medium with and without IFN-α or TNF-α for 14 days, or retained in unsupplemented TOM. (A) Representative phase-contrast images. Scale bars: 400 µm. (B) EVT marker mRNA expression measured by RT-qPCR. Bars represent mean log2 fold-change ± SD relative to untreated organoids in TOM (n=3 independent experiments). For all EVT conditions, EVT marker expression was significantly increased ($P < 0.001$) compared with the TOM condition (one-way ANOVA with Dunnett's multiple comparisons test).

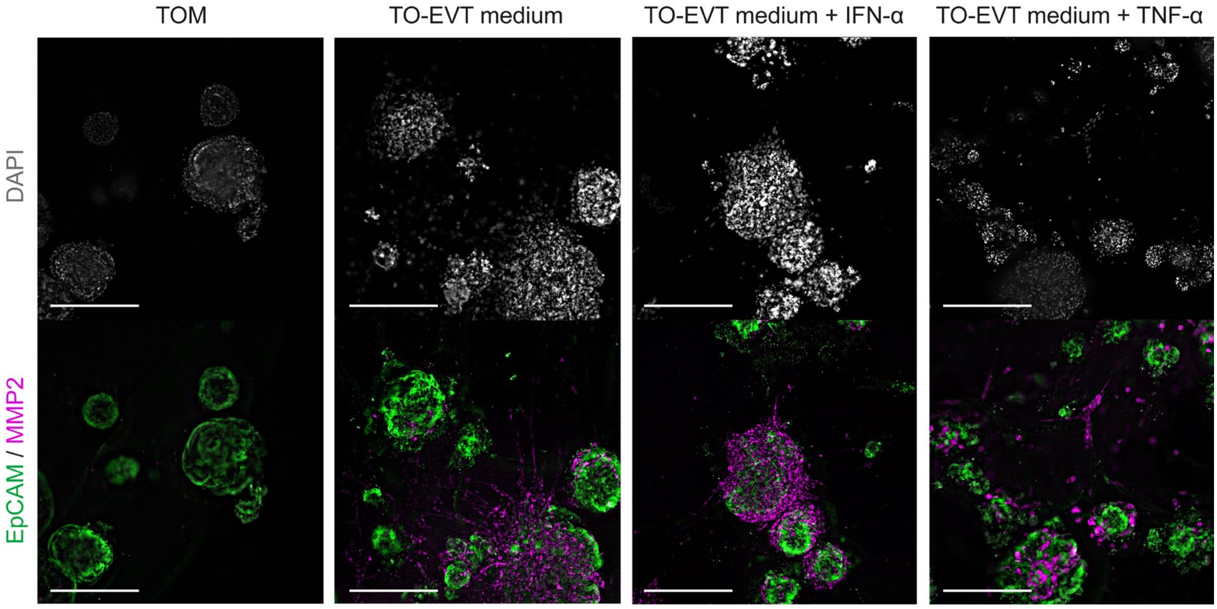

Fig. S4 (related to Fig. 2). Immunofluorescence images showing that IFN-α and TNF-α inhibit EVT invasion but do not affect EVT differentiation in trophoblast organoids. Organoids at EVT differentiation day 14 were stained for EpCAM (TSC marker, green), MMP2 (EVT marker, magenta), and DAPI (nuclei, white). Scale bars: 400 μm.

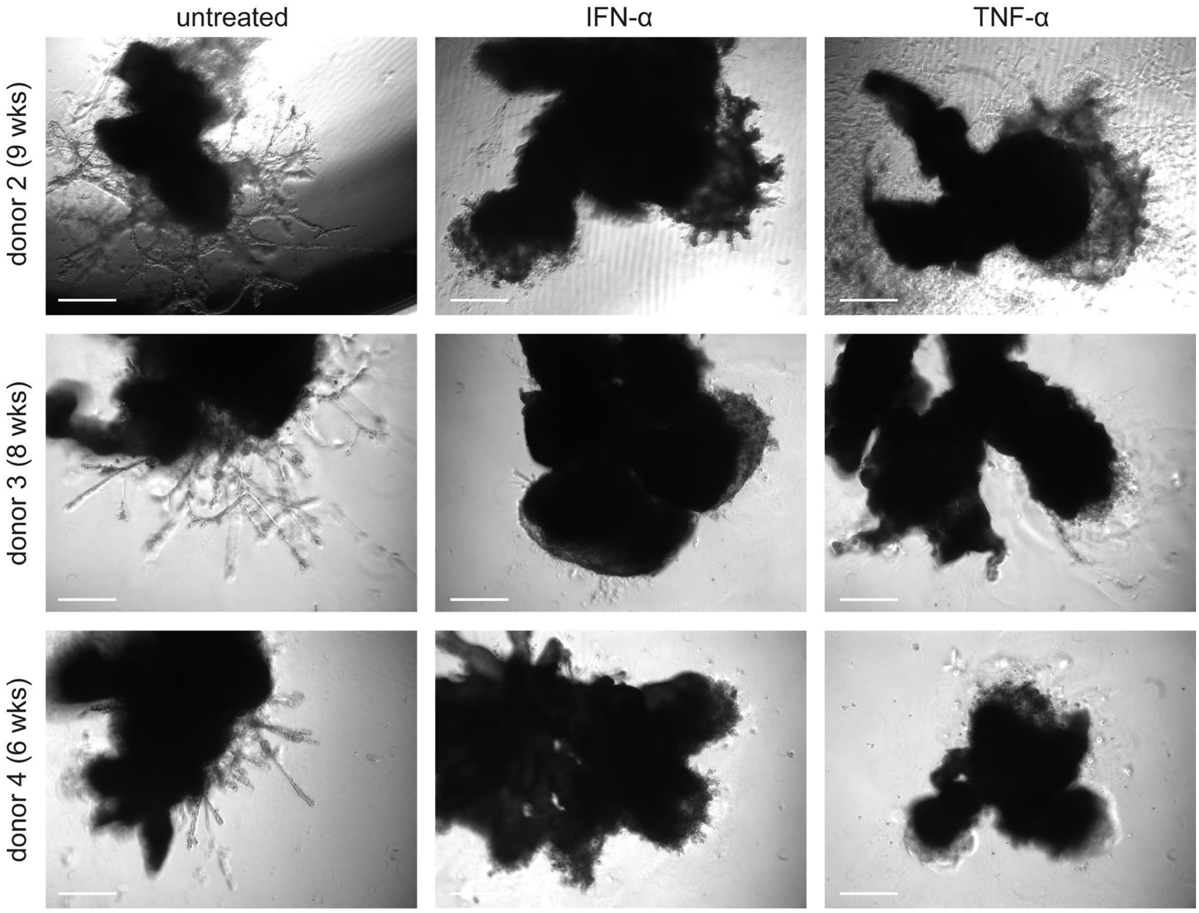

Fig. S5 (related to Fig. 3). Experiments with additional tissue donors showing that IFN-α and TNF-α affect EVT invasion in placental villous explants. Scale bars: 400 μm.

A

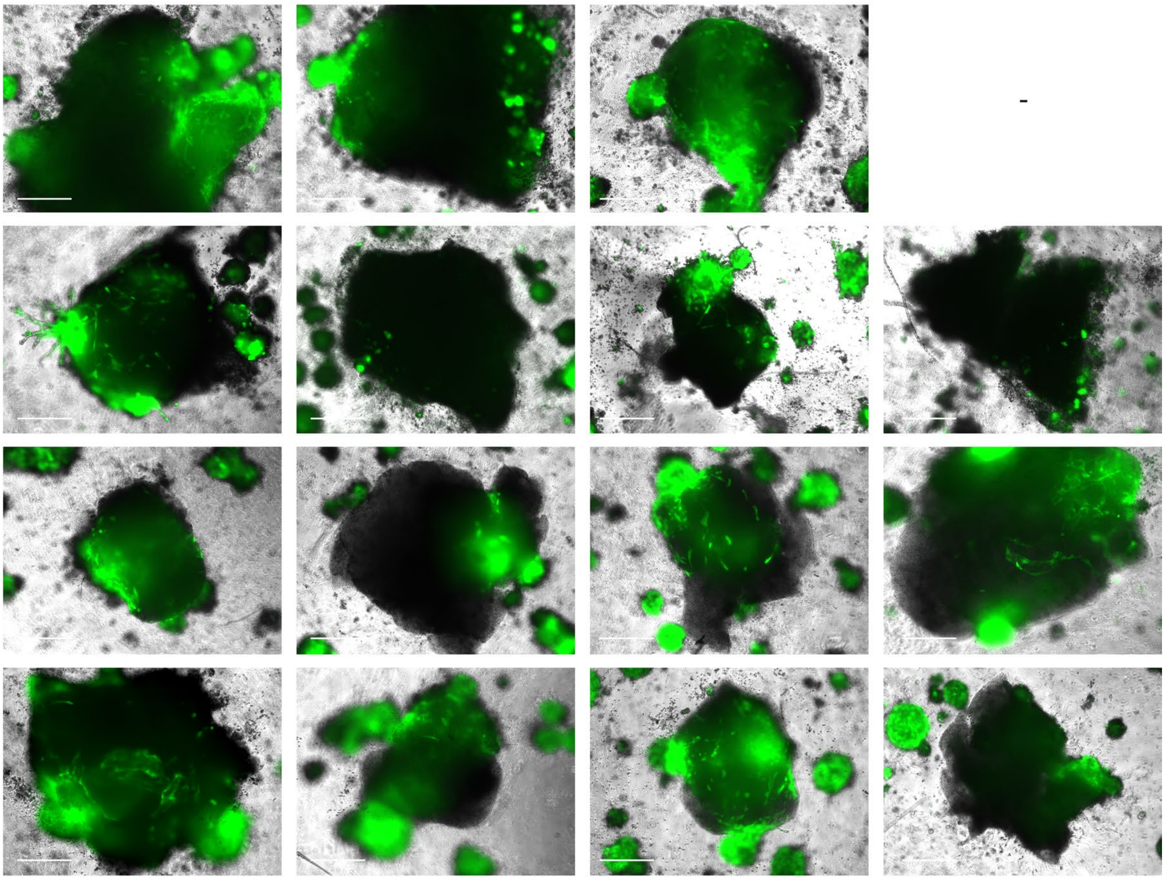

B

Invasion score		Example images
4	Very clear/deep invasion	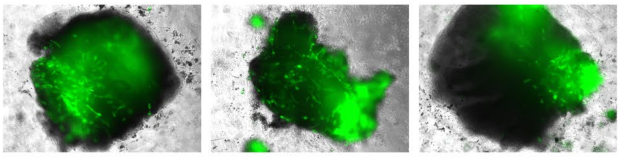
3	Clear invasion	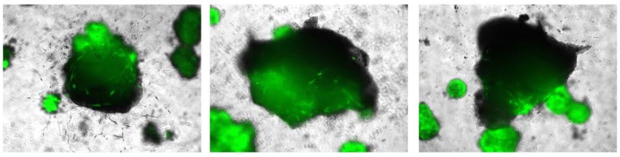
2	(Possibly) some invasion	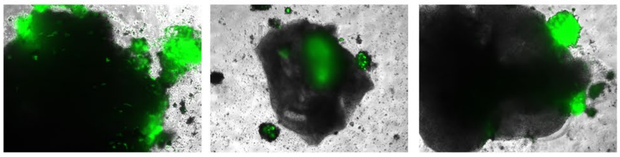
1	(Probably) no invasion	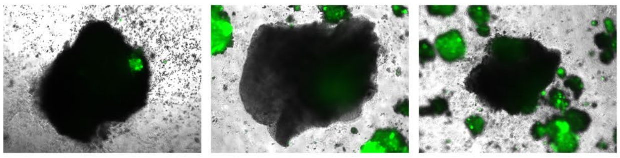

Fig. S6 (related to Fig. 5). Co-culture of GFP-positive trophoblast organoids and first-trimester decidua parietalis. (A) Experiments with additional tissue donors. Scale bars: 500 µm. (B) Invasion scoring table used to semi-quantify organoid-derived EVT invasion into decidual explants.

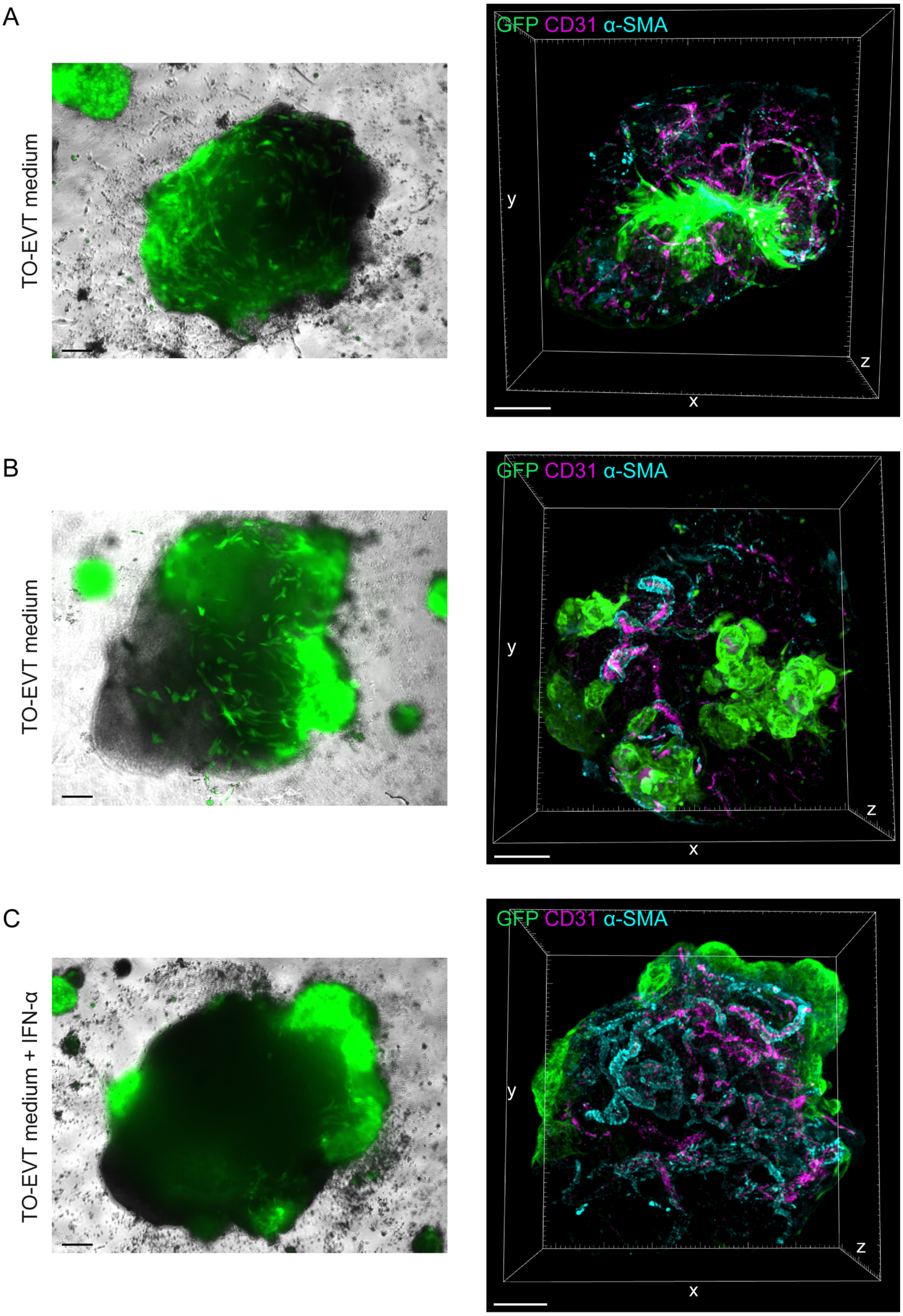

Fig. S7 (related to Fig. 6). Overview images of decidua parietalis co-cultured with GFP-positive trophoblast organoids. (A-C) Phase-contrast and immunostaining overview images corresponding to Fig. 6A-C, showing whole tissue fragments for contextualization purpose. The same phase-contrast images are included in Fig. 5B. Scale bars: 200 μm.

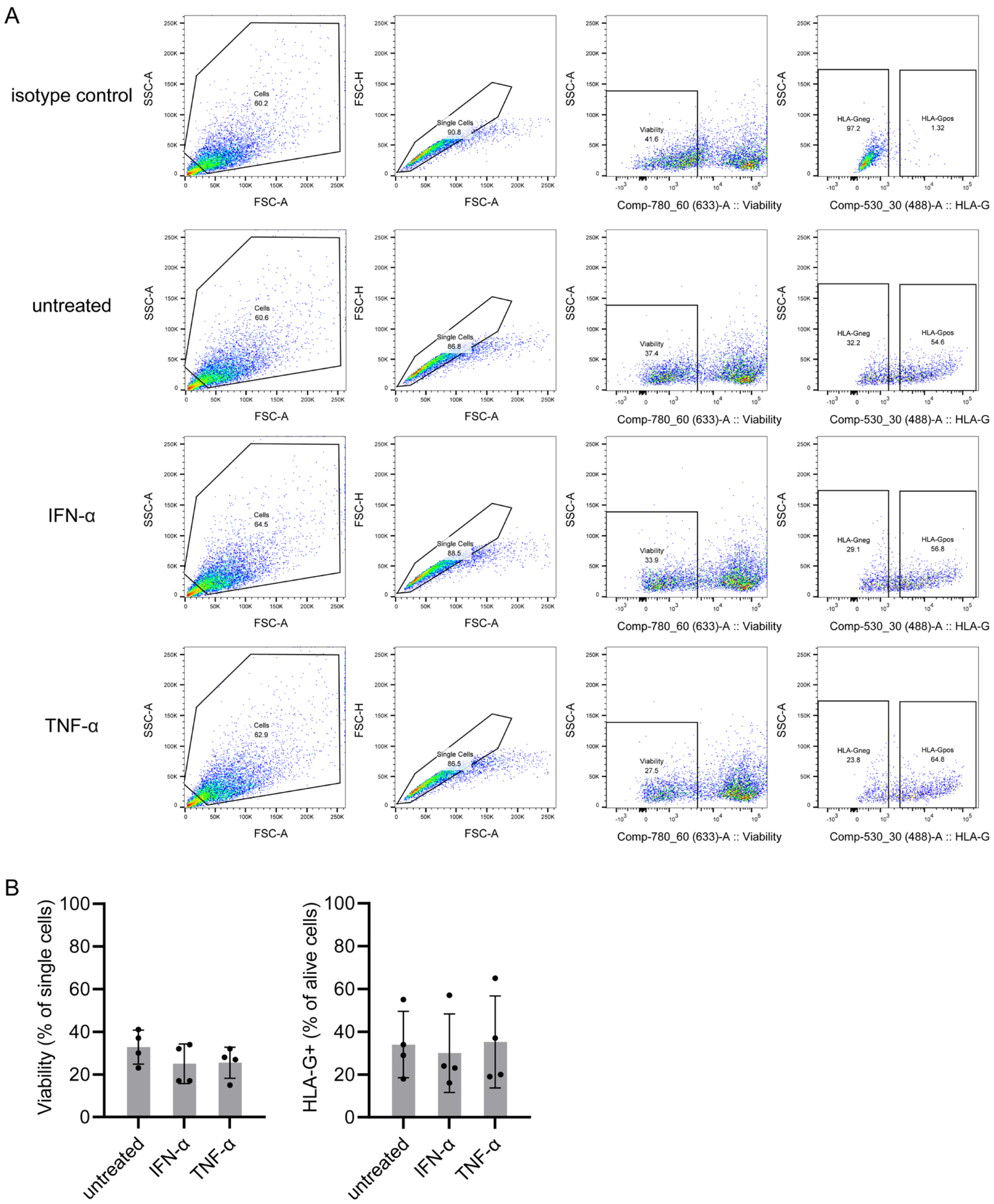

Fig. S8 (related to Fig. 7). Isolation of HLA-G-positive cells by FACS. (A) Gating strategy used. The images are representative for n=4 independent experiments. For the isotype control condition, a pool of untreated, IFN-α- and TNF-α-treated cells was used. (B) Percentages of viable and HLA-G-positive cells. Bars represent mean ± SD of n=4 independent experiments.

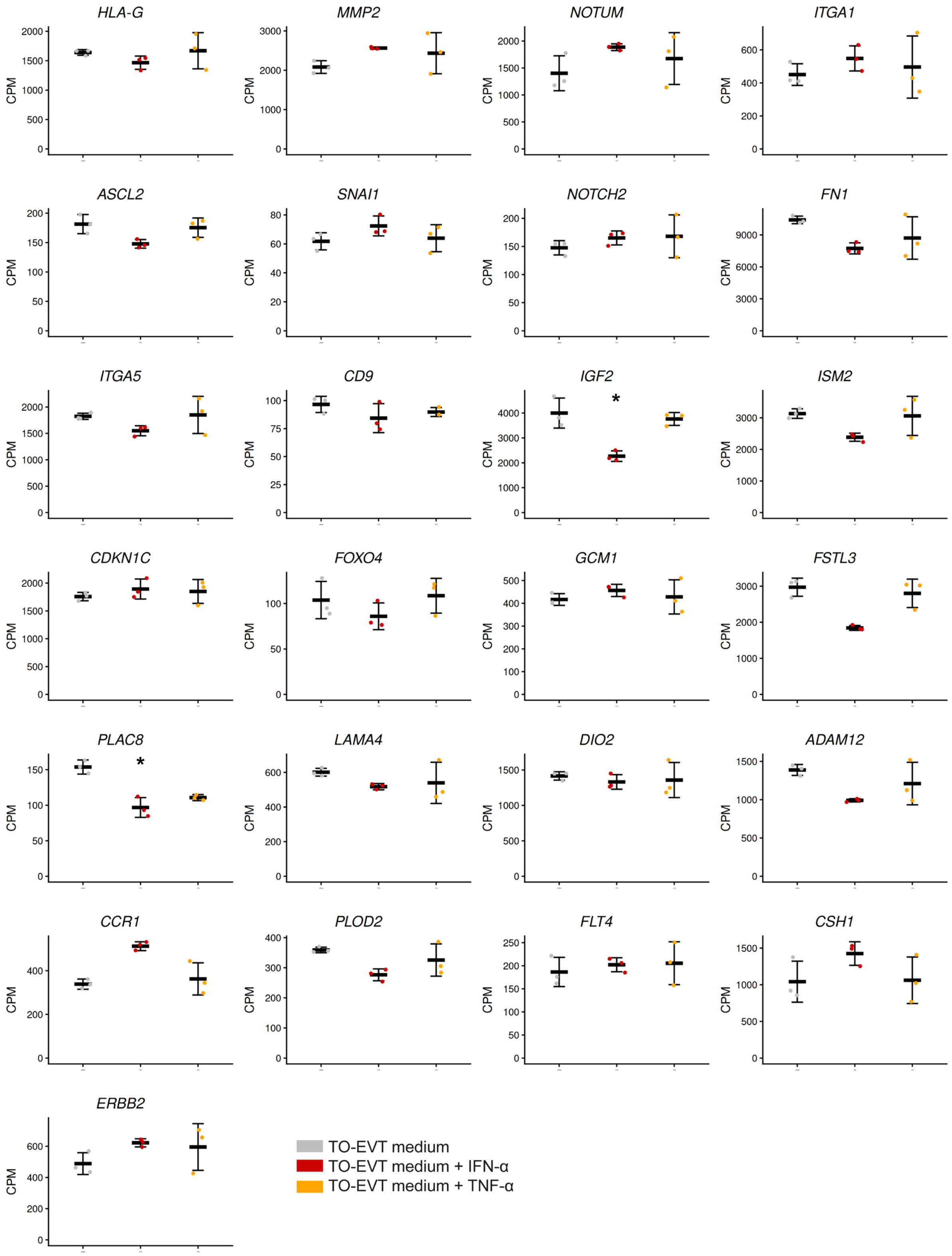

Fig. S9 (related to Fig. 7). EVT marker profile of organoid-derived EVTs after IFN-α and TNF-α exposure. Expression of EVT markers in transcriptome data of HLA-G-positive EVTs isolated from organoids treated with or without IFN-α/TNF-α, as batch-corrected counts per million (CPM). Graphs represent mean ± SD of n=3 independent experiments. *: FDR-adjusted $P < 0.05$.

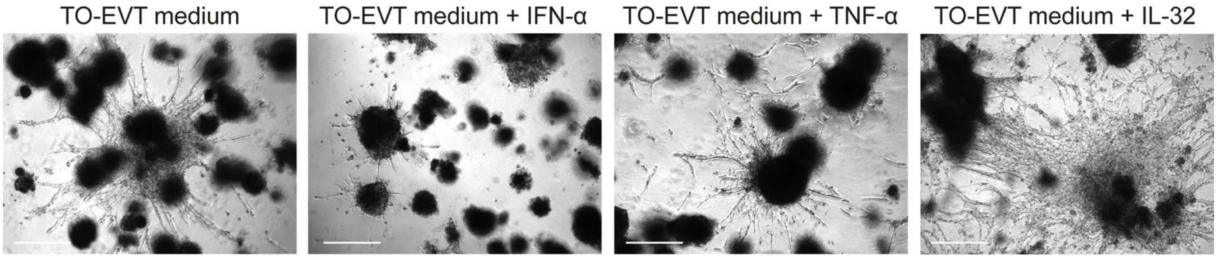

Fig. S10. IL-32 promotes EVT invasion, in contrast to IFN-α and TNF-α. Trophoblast organoids were induced to undergo EVT differentiation in TO-EVT medium for 14 days, in the presence and absence of IFN-α, TNF-α and IL-32. Representative phase-contrast images are shown of n=3 independent experiments. Scale bars: 500 µm.

SUPPLEMENTARY TABLES

Table S2 (related to Fig. 7). Differentially expressed genes upon IFN-α exposure that are involved in trophoblast invasion-associated enriched pathways. Differentially expressed genes (FDR-adjusted *P* < 0.05) in IFN-α-treated, organoid-derived EVTs versus untreated controls, sorted by log2 fold-change (FC).

Pathway	Symbol	Gene name	logFC	FDR
EPITHELIAL_MESENCHYMAL_TRANSITION	*IGFBP3*	insulin like growth factor binding protein 3	-2.461	1.23E-02
	SFRP1	secreted frizzled related protein 1	-1.558	7.25E-04
	COL1A2	collagen type I alpha 2 chain	-1.177	1.19E-02
	LOXL2	lysyl oxidase like 2	-1.065	1.45E-03
	HTRA1	HtrA serine peptidase 1	-0.964	4.01E-02
	LRRC15	leucine rich repeat containing 15	-0.807	2.77E-02
	GPC1	glypican 1	-0.588	3.84E-02
	COL4A1	collagen type IV alpha 1 chain	-0.572	2.43E-02
	SERPINE1	serpin family E member 1	0.424	4.23E-02
	PFN2	profilin 2	0.485	4.27E-02
	ANPEP	alanyl aminopeptidase, membrane	0.487	1.87E-02
	DAB2	DAB adaptor protein 2	0.544	3.55E-02
	NT5E	5'-nucleotidase ecto	1.354	3.65E-02
	COL11A1	collagen type XI alpha 1 chain	1.809	2.65E-03
HYPOXIA	*IGFBP3*	insulin like growth factor binding protein 3	-2.461	1.23E-02
	S100A4	S100 calcium binding protein A4	-1.240	4.11E-03
	CAV1	caveolin 1	-1.161	1.85E-02
	PLAC8	placenta associated 8	-0.655	2.33E-02
	GAA	alpha glucosidase	-0.619	1.85E-02
	HK2	hexokinase 2	-0.599	3.43E-02
	GPC1	glypican 1	-0.588	3.84E-02
	NDRG1	N-myc downstream regulated 1	-0.586	4.25E-02
	SLC2A3	solute carrier family 2 member 3	-0.502	4.90E-02
	GAPDH	glyceraldehyde-3-phosphate dehydrogenase	-0.500	2.23E-02
	LDHA	lactate dehydrogenase A	-0.494	4.90E-02
	GBE1	1,4-alpha-glucan branching enzyme 1	-0.482	1.22E-02
	BNIP3L	BCL2 interacting protein 3 like	-0.457	3.22E-02
	CITED2	Cbp/p300 interacting transactivator with Glu/Asp rich carboxy-terminal domain 2	-0.453	2.23E-02

	PDK1	pyruvate dehydrogenase kinase 1	-0.448	3.55E-02
	SERPINE1	serpin family E member 1	0.424	4.23E-02
GLYCOLYSIS	*IGFBP3*	insulin like growth factor binding protein 3	-2.461	1.23E-02
	CHST6	carbohydrate sulfotransferase 6	-1.762	2.20E-02
	EGLN3	egl-9 family hypoxia inducible factor 3	-1.050	3.55E-02
	HK2	hexokinase 2	-0.599	3.43E-02
	GPC1	glypican 1	-0.588	3.84E-02
	LDHA	lactate dehydrogenase A	-0.494	4.90E-02
	CITED2	Cbp/p300 interacting transactivator with Glu/Asp rich carboxy-terminal domain 2	-0.453	2.23E-02
	NT5E	5'-nucleotidase ecto	1.354	3.65E-02
	CACNA1H	calcium voltage-gated channel subunit alpha1 H	3.471	2.29E-02

Table S3 (related to Methods). Primer sets used for quantitative real-time PCR. *EEF2* and *GUSB* were used as reference genes.

Target gene symbol	Forward primer	Reverse primer	Amplicon size (bp)
CGB	CAGCATCCTATCACCTCCTGGT	CTGGAACATCTCCATCCTTGGT	102
EEF2	CTGGAGATCTGCCTGAAGGA	CGACCGGGTCAGATTTCTT	70
EPCAM	GCCAGTGTACTTCAGTTGGTGC	CCCTTCAGGTTTTGCTCTTCTCC	122
GUSB	GGAGTGCAAGGAGCTGGAC	ATTGAAGCTGGAGGGAACTG	145
HLA-G	CCACCACCCTGTCTTTGACTAT	ACGTCCTGGGTCTGGTCCT	114
ITGA1	CCGAAGAGGTACTTGTTGCAGC	GGCTTCCGTGAATGCCTCCTTT	107
MMP2	TGGCACCCATTTACACCTACAC	ATGTCAGGAGAGGCCCCATAGA	91
NOTUM	CTACTGGTGGAACGCAAACATGG	CGCACCACCTCCTGGATGATG	129
SDC1	CTATTCCCACGTCTCCAGAACC	GGACTACAGCCTCTCCCTCCTT	102
TEAD4	CAGGTGGTGGAGAAAGTTGAGA	GTGCTTGAGCTTGTGGATGAAG	120

Table S4 (related to Methods). Primary and secondary antibodies used for immunofluorescence.

Antibody	Manufacturer	Cat. no.	Source	Clone	Dilution	RRID
anti-goat IgG Alexa Fluor 647	Invitrogen	A21447	donkey	NA	1:1000	AB_2535864
anti-mouse IgG Alexa Fluor 488	Invitrogen	A21202	donkey	NA	1:1000	AB_141607
anti-mouse IgG Alexa Fluor 555	Invitrogen	A31570	donkey	NA	1:500	AB_2536180
anti-rabbit IgG Alexa Fluor 488	Invitrogen	A21206	donkey	NA	1:1000	AB_2535792
anti-rabbit IgG Alexa Fluor 555	Invitrogen	A31572	donkey	NA	1:500	AB_162543
α-SMA	Invitrogen	PA5-18292	goat	*polyclonal*	1:100	AB_10980764
CD31	Sigma-Aldrich	P8590	mouse	WM-59	1:200	AB_1078460
EpCAM	Cell Signaling Technology	2929	mouse	VU1D9	1:400	AB_2098657
EpCAM	Cell Signaling Technology	36746	rabbit	D4K8R	1:100	AB_2799105
GFP	Abcam	ab6556	rabbit	*polyclonal*	1:200	AB_305564
HLA-G	Exbio	11-291-C100	mouse	MEM-G/1	1:100	AB_10734353
MMP2	Cell Signaling Technology	40994	rabbit	D4M2N	1:200	AB_2799191

REFERENCES

1. van Voorden, A.J., Boussata, S., Keijser, R., Vermij, M., Wagner, M.K., Ganzevoort, W., and Afink, G.B. (2024). Generation of Bona Fide Human Induced Trophoblast Stem Cells by Direct Reprogramming of Term Umbilical Cord Cells. Int J Mol Sci *26*. 10.3390/ijms26010271.
2. van Voorden, A.J., Keijser, R., Veenboer, G.J.M., Lopes Cardozo, S.A., Diek, D., Vlaardingerbroek, J.A., van Dijk, M., Ris-Stalpers, C., van Pelt, A.M.M., and Afink, G.B. (2023). EP300 facilitates human trophoblast stem cell differentiation. Proc Natl Acad Sci U S A *120*, e2217405120. 10.1073/pnas.2217405120.
