# Supplementary material for: The pro-inflammatory cytokines IFN-α and TNF-α inhibit organoid-derived extravillous trophoblast invasion
Source: iScience. 2026 Jun 20;29(7):116431. doi: 10.1016/j.isci.2026.116431 (PMC13315761; doi:10.1016/j.isci.2026.116431)

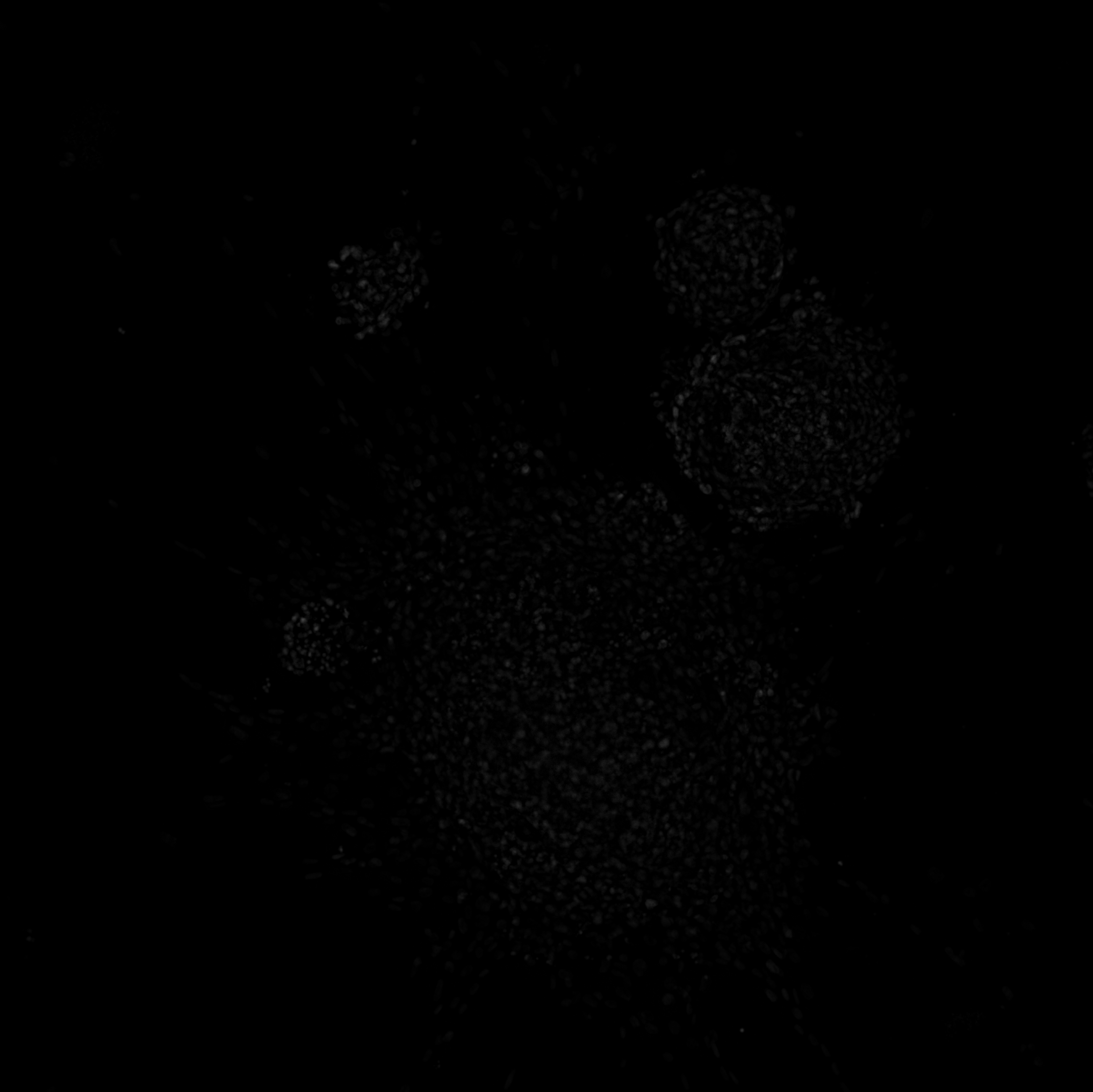

Supplement: Data S1. Raw Data-Fig2A_IFNA_DAPI_RawImageFile [file mmc3.pdf]
